# Intestine-specific Faf2 depletion ameliorates obesity and metabolic dysfunction-associated steatotic liver disease by impairing lipid absorption

**DOI:** 10.7150/ijbs.134379

**Published:** 2026-06-10

**Authors:** Jingjing Zhang, Norihiro Imai, Jinglei Cheng, Akiko Sugiyama, Hanna Kawecka, Dongming Liu, Michitaka Suzuki, Yuki Ohsaki, Shinya Yokoyama, Kenta Yamamoto, Takanori Ito, Keiko Maeda, Yoji Ishizu, Takashi Honda, Tetsuya Ishikawa, Michał Woźniak, Hiroaki Wake, David E. Cohen, Hiroki Kawashima

**Affiliations:** 1 Department of Gastroenterology and Hepatology, Nagoya University Graduate School of Medicine, Aichi, Japan.; 2 Department of Anatomy and Molecular Cell Biology, Nagoya University Graduate School of Medicine, Aichi, Japan.; 3 Karsh Division of Gastroenterology & Hepatology, Cedars-Sinai Medical Center, CA, USA.; 4 Department of Medical Chemistry, Medical University of Gdańsk, Gdańsk, Poland.; 5 Department of Hepatobiliary Cancer, Liver Cancer Research Center, Tianjin Medical University Cancer Institute and Hospital, Tianjin, China.; 6 Department of Anatomy and Histology, Fukushima Medical University School of Medicine, Fukushima, Japan.; 7 Division of Cell and Tissue Morphology, Department of Anatomy, Sapporo Medical University School of Medicine, Hokkaido, Japan.

**Keywords:** liver steatosis, obesity, Faf2, lipid metabolism, chylomicron

## Abstract

Fas-associated factor family member 2 (Faf2) is an endoplasmic reticulum (ER)-associated protein implicated in apolipoprotein B (ApoB) metabolism, yet its physiological role in the intestine remains unclear. To investigate this, we generated intestine-specific Faf2 knockout (Faf2-IKO) mice and fed them either a normal diet or a high-fat diet (HFD). Faf2-IKO mice exhibited reduced body weight and adiposity under both conditions, improved glucose tolerance, and protection against HFD-induced hepatic steatosis. In the intestine, Faf2 deficiency was associated with lipid droplet accumulation, intracellular retention of ApoB48, and reduced chylomicron abundance in lacteals, consistent with impaired intestinal lipoprotein handling. Faf2-IKO mice also exhibited elongation of the small intestine and villi after weaning. Despite these changes, Faf2-IKO mice showed elevated postprandial triglyceride and free fatty acid levels during lipid tolerance tests, indicating paradoxical alterations in postprandial lipid responses. Additionally, Faf2 deficiency was associated with ER dilation and increased expression of genes associated with ER-related cellular responses. Furthermore, in the livers of Faf2-IKO mice, genes that govern cholesterol homeostasis and sterol biosynthetic pathways were significantly enriched. Collectively, these findings suggest that intestinal Faf2 deficiency alters chylomicron-associated lipid handling and is associated with protection against obesity and metabolic dysfunction-associated steatotic liver disease in mice.

## 1. Introduction

Fas-associated factor family member 2 (Faf2), also known as UBXD8 or ETEA, encodes a multifunctional protein that localizes to the endoplasmic reticulum (ER) and lipid droplet (LD), where it has been implicated in the regulation of lipid metabolism and protein quality control 1-4. Recent studies further suggest that Faf2 participates in the regulation of lipid-associated organelles, including the ER, LD, and peroxisomes, as well as in cellular responses to lipid stress 5-6. Together, these findings position Faf2 as a potential coordinator of lipid metabolism and organelle homeostasis.

Apolipoprotein B (ApoB) provides the structural scaffold for triglyceride (TG)-rich lipoproteins, enabling their assembly and secretion in both liver and intestine. In hepatocytes, ApoB100 (and ApoB48 in mice) drives very-low-density lipoprotein (VLDL) assembly, a process tightly coupled to TG availability and controlled by ER-associated degradation and autophagy 7-9. In contrast, enterocytes rely on ApoB48 to assemble chylomicrons (CM) for lymphatic export 10. Properly lipidated ApoB-containing lipoproteins undergo intracellular transport and secretion. In enterocytes, this process involves pre-chylomicron transport vesicles, which mediate ER-to-Golgi export and are essential for CM secretion 11. Conversely, when lipid supply is limited or ApoB folding is impaired, ApoB is directed toward ER-associated degradation or post-ER proteolysis, with autophagy contributing to its terminal clearance.

Despite this framework, the mechanisms regulating ApoB48 handling and CM secretion in enterocytes remain incompletely understood. In hepatocytes, Faf2 has been implicated in ApoB turnover and the regulation of VLDL production 2,3. However, whether intestinal Faf2 regulates ApoB48-containing lipoprotein handling through similar or distinct mechanisms remains unclear. Given the complex requirements for CM biogenesis and secretion, altered ApoB lipidation, intracellular trafficking, ER stress, mechanistically, microsomal triglyceride transfer protein (MTTP)-dependent lipoprotein handling or disruption of organelle homeostasis could each influence ApoB48 processing and CM export in enterocytes.

To investigate the role of intestinal Faf2 in nutrient handling and systemic energy homeostasis, we generated intestine-specific Faf2 knockout (Faf2-IKO) mice. We found that intestinal Faf2 deficiency was associated with intracellular ApoB48 accumulation, reduced CM abundance in intestinal lacteals, LD retention, and structural alterations of the ER in enterocytes. These changes coincided with improved glucose tolerance under overnutrition and attenuated hepatic steatosis (HS). Together, these findings suggest that intestinal Faf2 contributes to enterocyte lipid handling and ER-associated organelle homeostasis and may represent a potential therapeutic target for obesity and metabolic dysfunction-associated steatotic liver disease.

## 2. Methods

### 2.1. Animals and diets

C57BL/6J mice (Jackson Laboratory, Bar Harbor, ME, USA; Stock #000664) served as the wild-type (WT) controls. C57BL/6J background mice with *Faf2* flanked by two *LoxP* sites (*Faf2^flox/flox^*) were crossed with mice expressing Cre recombinase driven by the *Villin 1* promoter (hereafter called *Villin-Cre* mice) to generate intestine-specific deletion of *Faf2* knockout mice (Faf2-IKO) (The Jackson Laboratory, *Villin-Cre*; Stock #004586) 12. Although Villin-Cre mediates recombination during embryonic development, phenotypic and functional analyses in the present study were conducted exclusively in postnatal and adult mice 13,14. All the mice were viable and showed no detectable developmental defects under standard housing conditions. Genotyping was performed using genomic DNA extracted from the ear punch tissue. PCR amplification was conducted using specific primers targeting the *Faf2* allele and the *Villin-Cre* transgene (Table S1). Animals were maintained on a 12-hour light/dark cycle with *ad libitum* access to water and food in a barrier facility with sentinel monitoring, stringent biosecurity, daily health checks, and veterinary oversight. Male and female mice were weaned at four weeks of age and fed a normal diet (ND) (CLEA Rodent Diet CE-2: crude protein 24.96%, crude fat 4.76 %, crude fiber 4.84 %, NFE 50.54 %, energy 3.45 kcal/g; CLEA Japan, Tokyo, Japan) for 12 weeks. Alternatively, 4-week-old male and female mice were fed a high fat diet (HFD) (D12492: 20% kcal from protein, 60% kcal from fat, and 20% kcal from carbohydrate; energy density 5.21 kcal/g; Research Diets Inc., New Brunswick, NJ, USA) for 12 weeks. In total, 169 mice were used in this study. Male and female mice were subjected to *in vivo* phenotyping experiments, including body weight (BW) measurements, food intake quantification, behavioral observations, metabolic phenotyping, glucose tolerance tests, insulin tolerance tests, lipid tolerance tests (LTT), and histological analyses of the liver, adipose tissues, and intestinal epithelium. Data from male and female mice were analyzed independently and are presented separately, unless otherwise stated. Additional mechanistic and molecular analyses were performed in male mice, including plasma lipoprotein profiling through high-performance liquid chromatography (HPLC), RNA-sequencing (RNA-seq), jejunal qPCR, and transmission electron microscopy. The number of animals used in each experiment is provided in the corresponding figure legends. At endpoint, mice were euthanized and blood collected by cardiac puncture for serum isolation; tissues were processed immediately or flash-frozen in liquid nitrogen and stored at -80 °C. All animal experiments were performed in accordance with the protocols approved by the Institutional Animal Care and Use Committee of Nagoya University Graduate School of Medicine. The enhanced green fluorescent protein (eGFP) knock-in mice were generated with the support of the Advanced Research Support Platform for Model Animals.

### 2.2. Tissue sample preparation and histological analysis

The dissected tissues were fixed with 4% PFA in PBS for 24 hours at 4 °C. Samples were embedded in paraffin and sectioned at a 3 μm thickness for liver and 5 μm for epididymal white adipose tissue (eWAT, male), periovarian white adipose tissue (pWAT, female) and jejunum. Hematoxylin and eosin (H&E) staining was performed according to standard protocols. Histological images were captured using a BZ-9000 microscope (Keyence, Osaka, Japan). For all histological assessments, quantitative analyses were performed using ImageJ software (National Institutes of Health, Bethesda, MD, USA).

HS was quantified as the percentage of LD-positive areas relative to the total parenchymal area. For each mouse, at least five randomly selected nonoverlapping fields were analyzed. In white adipose tissue, adipocyte size was determined by measuring the cross-sectional area of individual adipocytes from H&E-stained sections, with ≥50 adipocytes analyzed per mouse from at least three representative fields. The villus height and crypt depth in the small intestine were measured in H&E-stained jejunal sections. For male mice, more than 20 villi and 48 crypts were analyzed per mouse, whereas for female mice, more than 18 villi and 18 crypts were analyzed per mouse.

### 2.3. Metabolic monitoring

Energy balance parameters, including oxygen consumption (VO_2_), carbon dioxide production (VCO_2_), respiratory exchange ratio (RER), energy expenditure (EE), locomotor activity, and food intake, were assessed using a metabolic monitoring system. Twelve-week-old mice were housed in individual cages for acclimatization prior to metabolic monitoring. The mice were then housed in temperature-controlled (23 °C ± 0.2 °C) cabinets with a 12-hour light/dark cycle and monitored using the Promethion Metabolic Screening System (Sable Systems International, North Las Vegas, NV, USA). Rates of VO_2_ and VCO_2_ were determined at 5-min intervals. The RER values were calculated as VCO_2_/VO_2_. After 1 day acclimatization, metabolic parameters were recorded over 6 days. Physical activity was determined based on the beam breaks within a grid of photosensors built outside the cages. EE was calculated using indirect calorimetry. Fecal caloric content was measured using bomb calorimetry 15,16.

### 2.4. Glucose tolerance test

Fourteen-week-old male and female HFD-fed mice were fasted for 6 hours and then intraperitoneally injected with glucose (D-(+)-glucose; Sigma-Aldrich, St. Louis, MO, USA) at a dose of 1.0 g/kg BW 17. Blood samples were obtained from the tail vein at baseline (0 min) and 15, 30, 60, 90, and 120 min after glucose administration. The glucose levels were measured immediately using a handheld glucometer (Medisafe fit, Terumo Corp., Tokyo, Japan).

### 2.5. Insulin tolerance test

Fifteen-week-old male and female mice fed a HFD were fasted for 4 hours and then intraperitoneally injected with insulin at a dose of 0.5 U/kg BW 18. Blood was collected from the tail vein at baseline (0 min) and 15, 30, 60, 90, and 120 min after the injection of insulin (Eli Lilly Japan). The glucose levels were measured immediately using a glucometer (Medisafe fit, Terumo Corp., Tokyo, Japan).

### 2.6. LTT

For LTT, eleven-week-old male and female mice maintained on a ND were fasted for 16 hours, followed by oral gavage of olive oil (10 μl/g BW) 19,20. Blood samples (50 μl) were collected from the tail vein at baseline (0 min) and 1, 2, and 3 hours post-gavage. Samples were centrifuged at 1,000 × *g* for 20 minutes at 4 °C to obtain serum. After centrifugation, TG and free fatty acid (FFA) concentrations in the plasma samples were measured using the Wako L-Type TG M test (FUJIFILM, MA, USA) and Wako HR series NEFA-HR (2) ((FUJIFILM, MA, USA), respectively.

### 2.7. Western blot

Protein concentrations were determined prior to electrophoresis. For immunoblotting, 30 μg of total protein per lane was loaded for detection of Faf2 and eGFP. Corresponding β-Actin loading controls were obtained from separate membranes prepared using identical protein lysates and loading amounts. For detection of ApoB, 75 μg of total protein per lane was loaded, and β-Actin and HSP proteins were detected in parallel at the same loading amount for ApoB, reflecting differences in protein abundance and detection sensitivity. Proteins were separated on Bolt Bis-Tris Plus gels, followed by transfer onto nitrocellulose membranes. In selected experiments, membranes were briefly stained with Ponceau S (Cat #A40000279; Thermo Scientific) solution (0.1% Ponceau S in 5% acetic acid) to verify uniform protein transfer and loading and then destained with distilled water prior to blocking. Membranes were subsequently blocked with 5% nonfat dry milk in TBST (TBS containing 0.05% Tween-20) for 1 hour at room temperature, then incubated with primary antibodies (Table S2) overnight at 4 °C. After washing, membranes were incubated with appropriate secondary antibodies (Table S2), including horseradish peroxidase (HRP)-conjugated anti-rabbit IgG for ApoB and Faf2 detection and StarBright Blue 520 anti-mouse IgG for HSP detection. β-Actin was detected using a rhodamine-conjugated primary antibody without secondary antibody incubation. Protein signals were visualized using enhanced chemiluminescence or fluorescence detection as appropriate.

### 2.8. Quantitative PCR

Relative mRNA expression was determined by qPCR using the SYBR Green Real-Time PCR Master Mix (Applied Biosystems, Foster City, CA, USA). Total RNA was isolated from the small intestine using the RNeasy Fibrous Tissue Mini Kit (QIAGEN, Hilden, Germany), following the manufacturer's instructions. cDNA was synthesized using the High-Capacity cDNA Reverse Transcription Kit (Applied Biosystems, Foster City, CA, USA). Equal amounts of mRNA were subjected to qPCR using the QuantStudio 6 Flex Real-Time PCR System (Applied Biosystems, Foster City, CA, USA) in a 384 plate. Gene expression levels were normalized to those of cyclophilin A, which was selected as the reference gene based on its validated expression stability under metabolic conditions 21. For selected targets, β-Actin was also used as a secondary reference gene for confirmation. The sequences of the oligonucleotides used for qPCR are listed in Table S3.

### 2.9. Transmission electron microscopy

Sixteen-week-old male mice maintained on a ND were fasted for 16 hours, gavaged with olive oil (10 μL/g BW), and jejunal tissues were collected 2 hours later and immediately fixed overnight in 2.5% glutaraldehyde and 2% paraformaldehyde in 0.1 mol/l sodium cacodylate buffer (pH 7.4).Samples were post-fixed in 1% osmium tetroxide and 0.1% potassium ferrocyanide in the same buffer, dehydrated using a graded ethanol series, and embedded in Quetol-812 resin. Ultrathin sections were counterstained with uranyl acetate and lead citrate, and examined using a JEM-1400PLUS transmission electron microscope (JEOL, Tokyo, Japan). Images were acquired using a transmission electron microscope at magnifications of ×8,000-×30,000. For each mouse, at least 10 non-overlapping fields were analyzed from two independent ultrathin sections, and each group included three mice. Fields were randomly selected from areas containing intact enterocytes with well-preserved ultrastructure without prior knowledge of the genotype. Quantification was performed by an investigator blinded to the experimental groups.

### 2.10. Biochemical analysis of blood samples

Blood samples were collected from sixteen-week-old mice via cardiac puncture at the time of sacrifice under anesthesia in a non-fasting state. Blood collection was performed at approximately 17:00. No anticoagulant was used, and samples were allowed to clot at 22-25 °C for 30 min, followed by incubation at 4 °C overnight, before centrifugation at 3,000 × g for 30 min at room temperature to obtain serum. Serum concentrations of TG, phospholipids, FFA, and total cholesterol were measured by SRL, Inc. (Tokyo, Japan). Serum lipoprotein profiles were analyzed by gel filtration HPLC using the LipoSEARCH system (Immuno-Biological Laboratories, Gunma, Japan) 22,23.

### 2.11. Immunofluorescence staining

Immunofluorescence staining was performed on frozen ileal sections of the small intestine. Ileal tissues were fixed in 4% PFA for 48 hours at 4 °C and cryoprotected in 30% sucrose at 4 °C until fully infiltrated, embedded, and cryosectioned. PFA-fixed frozen slides were washed and rehydrated in TBS. Sections were permeabilized with 0.05% Triton X-100 for 10 minutes at room temperature, followed by three washes in TBS. Non-specific binding was blocked with 3% bovine serum albumin for 1 hour at room temperature. Sections were then incubated overnight at 4 °C with primary antibodies (Table S2) diluted in 3% BSA, washed once in TBS, twice in TBST (TBS containing 0.05% Tween-20), and once in TBS, followed by incubation with fluorophore-conjugated secondary antibodies (Table S2) for 90 min at 22-25 °C. Nuclei were counterstained with Hoechst 33342 (Cat#H3570; Invitrogen, Carlsbad, CA, USA). Images were acquired using a Zeiss LSM 880 confocal laser-scanning microscope (Zeiss, Jena, Germany).

### 2.12. Immunohistochemistry

Ki67 immunohistochemistry was performed on paraffin-embedded intestinal sections. Briefly, intestinal tissues were fixed in 4% PFA, embedded in paraffin, and sectioned at 4 μm. After deparaffinization and rehydration, antigen retrieval was performed in 10 mM citrate buffer (pH 6.0) using the heat-induced epitope retrieval method. Endogenous peroxidase activity was quenched with 0.3% hydrogen peroxide, and non-specific binding was blocked with 5% BSA. Sections were incubated overnight at 4 °C with an anti-Ki67 primary antibody (Table S2), followed by incubation with an HRP-conjugated secondary antibody (Table S2). Immunoreactivity was visualized using a DAB substrate and counterstained with hematoxylin. Ki67-positive cells within the intestinal crypt region were assessed for quantification. For each mouse, six randomly selected sections were analyzed, and the number of Ki67-positive cells was normalized to the total number of crypt epithelial cells. All analyses were performed in a blinded manner.

### 2.13. RNA-seq

Total RNA was extracted from the liver and skeletal muscle (SM) tissues of HFD-fed Faf2-IKO mice and their littermate controls using the RNeasy Mini Kit (QIAGEN, Hilden, Germany) for the liver and the RNeasy Fibrous Tissue Mini Kit (QIAGEN, Hilden, Germany) for SM, following the manufacturer's instructions. RNA integrity was evaluated using an Agilent 2100 Bioanalyzer, and only samples with an RNA integrity number (RIN) greater than 8 were retained for subsequent transcriptomic analyses. Sequencing libraries were constructed using the TruSeq Stranded mRNA Library Prep Kit (Illumina, San Diego, CA, USA) and sequenced on an Illumina NovaSeq 6000 platform, generating 150-bp paired-end reads, with approximately 40 million reads per sample. Raw sequencing data were subjected to quality assessment using FastQC and adapter trimming using the Trimmomatic software. High-quality reads were aligned to the murine reference genome (GRCm38/mm10) using HISAT2, and gene-level read counts were quantified using featureCounts. Differential gene expression analysis was performed using the DESeq2 package in R, applying a significance threshold of |log2 fold-change| > 2 and an adjusted p-value < 0.05. Visualization of the results included volcano plots (generated with EnhancedVolcano) and heatmaps (constructed using pheatmap). Functional enrichment was illustrated using bubble plots (ggplot2) and protein-protein interaction networks (Cytoscape). Gene Set Enrichment Analysis was conducted using the fgsea package with gene sets from MSigDB (HALLMARK and KEGG collections). The significance was evaluated based on the normalized enrichment score and false discovery rate.

### 2.14. Statistical analyses

**Figure 1:**
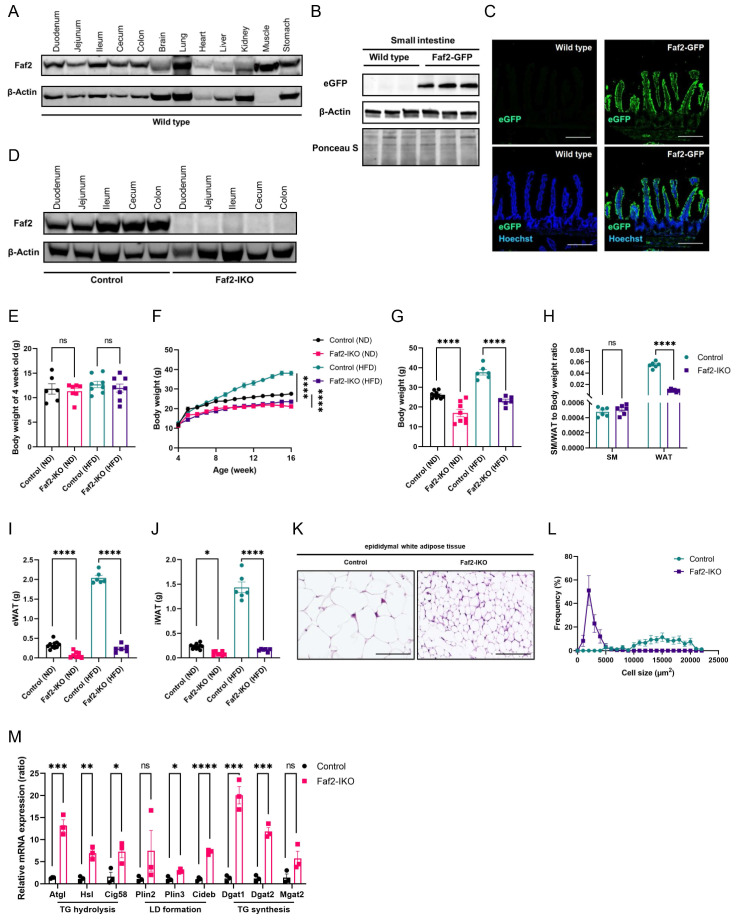
Intestine-specific Faf2 depletion mice display reduced body weight and resistance to HFD-induced obesity. 4-week-old male Faf2-IKO and littermate mice were fed a ND or HFD for 12 weeks. (A) Faf2 protein expression across major tissues was analyzed by western blot, with β-Actin used as a loading control. (B) The expression of eGFP in the small intestine was examined by western blot. β-Actin was used as a loading control, and Ponceau S staining was employed as an additional control for the western blot detecting eGFP in small-intestine lysates from Wild type and Faf2-GFP knock-in mice. (C) Immunofluorescence staining of the ileum in the small intestine of Faf2-GFP mice revealed colocalization of native eGFP expression. Green = native eGFP, blue = Hoechst. Scale bars: 200 µm (D) Western blot analysis of intestinal tissue confirmed Faf2 expression, with β-Actin serving as a loading control. (E-G) Body weight at 4 weeks of age (E), body weight curves (F), and adult body weight (G) of male mice fed a ND or HFD. (n = 6,10 for WT ND, 7,8 for Faf2-IKO ND, 6 for WT HFD, 6 for Faf2-IKO HFD). (H) SM-to-body weight ratio and WAT-to-body weight ratio of male mice fed the HFD (n = 6 per group). (I, J) eWAT (I) and iWAT (J) weights in male mice fed a ND or HFD (n = 10 for WT ND, 8 for Faf2-IKO ND, 6 for WT HFD, 6 for Faf2-IKO HFD). (K, L) H&E staining (K) and adipocyte size (L) in eWAT of male mice fed a HFD (n = 6 per group). Scale bars: 100 µm. (M) Quantitative PCR analysis of mRNA expression of adipose lipolysis-related genes in iWAT from ND-fed male Control and Faf2-IKO mice (n = 3 per group). β-Actin was used as the internal reference gene. Data are shown as mean ± SEM. ns: not significant, *p<0.05, **p<0.01, ***p<0.001, ****p<0.0001. Statistical analyses were performed using one-way ANOVA followed by Bonferroni post-hoc testing for panels E, G, I, and J; two-way ANOVA followed by Bonferroni post-hoc testing for panel F; and multiple unpaired two-tailed Student's t-tests without correction for multiple comparisons for panels H and M. Abbreviations: eGFP, enhanced green fluorescent protein; eWAT, epididymal white adipose tissue; Faf2, fas-associated factor family member 2; Faf2-IKO, intestine-specific Faf2 knockout; H&E, hematoxylin and eosin; HFD, high-fat diet; iWAT, inguinal white adipose tissue; LD, lipid droplet; ND, normal diet; SM, skeletal muscle; TG, triglycerides; WT, wild type.

Data are presented as mean values with error bars representing the standard error of the mean. For comparisons between two groups with a single factor, a two-tailed unpaired Student's t-test was used. For gene expression analyses, each gene was analyzed independently using a two-tailed unpaired Student's t-test without correction for multiple comparisons, as each gene represents a distinct biological hypothesis. For time-course experiments (e.g., glucose tolerance test (GTT), insulin tolerance test (ITT), and LTT), two-way ANOVA was performed with genotype and time as independent variables, followed by Bonferroni post-hoc testing. For comparisons among multiple groups with a single factor (e.g., as in Figure 1E), one-way ANOVA followed by Bonferroni post-hoc testing was applied. For experiments involving two independent variables, two-way ANOVA followed by Bonferroni post-hoc testing was used. For metabolic cage analyses, ANOVA was used for mass-independent variables including pedestrian locomotion, total distance in the cage, RER, local activity and energy balance, whereas ANCOVA was used for mass-dependent variables, including VO_2_, VCO_2_, EE, food intake and water intake, on metabolic parameters across light/dark cycles. Differences were considered statistically significant at p < 0.05. Figures were prepared using color-blind-safe color palettes, with sex information clearly indicated in the figure legends. All analyses were performed using GraphPad Prism 10 (GraphPad Software, Boston, MA, USA) and R statistical software (R Core Team, R Foundation for Statistical Computing, Vienna, Austria).

## 3. Results

### 3.1. Depletion of intestine-specific Faf2 reduces BW and resistance to HFD-induced obesity in mice

Faf2 is broadly expressed in multiple organs, including the brain, lung, heart, liver, intestine, muscle and kidneys (Fig. 1A). However, because of its nature as a non-membrane-spanning protein, Faf2 is highly prone to proteolytic degradation, making *in vivo* detection technically challenging 24. To overcome this, we used the CRISPR-Cas9 system to insert eGFP tag at the C-terminus of the endogenous Faf2 locus, thereby generating a Faf2-GFP knock-in mouse model. In these mice, Faf2 expression within the intestine was predominantly localized to the villi (Fig. 1B, C). To investigate the functional role of intestinal Faf2 in lipid metabolism, we established Faf2-IKO mice, thereby deleting Faf2 specifically in intestinal epithelial cells (Fig. 1D). All knockout mice generated in this study were viable until adulthood under standard housing conditions and exhibited normal mating behavior and fertility. The BW at the time of weaning (4 weeks of age) did not differ between male or female control and Faf2-IKO mice (Fig. 1E; Fig. S1A). In contrast, compared with littermate controls, Faf2-IKO mice of both sexes fed either ND or HFD exhibited a significantly lower adult BW (Fig. 1F, G; Fig. S1B, C). These findings indicate that the reduction in BW emerged after weaning. Although the ratios of SM-to-BW did not differ, the ratios of WAT-to-BW were reduced in Faf2-IKO male and female mice compared to control mice fed a HFD (Fig. 1H; Fig. S1D). Moreover, fat mass, including eWAT and inguinal white adipose tissue (iWAT) weight was reduced in males Faf2-IKO mice under both ND and HFD conditions (Fig. 1I, J). In female mice, pWAT and iWAT weight were reduced in Faf2-IKO mice under HFD conditions (Fig. S1E, F). In line with these findings, adipocyte size was significantly diminished in the eWAT of male mice and pWAT of female mice (Fig. 1K, L; Fig. S1G, H). At the molecular level, the expression of key lipolytic genes, such as *Atgl*, *Hsl*, and *Cgi58*, was significantly upregulated in the iWAT of both male and female Faf2-IKO ND fed mice (Fig. 1M; Fig. S1I). These results indicate that intestinal Faf2 deficiency reduces BW and adiposity and is associated with resistance to HFD-induced obesity in mice.

### 3.2. Deficiency of intestine-specific Faf2 protects mice from HFD-induced HS and improves glucose tolerance

**Figure 2:**
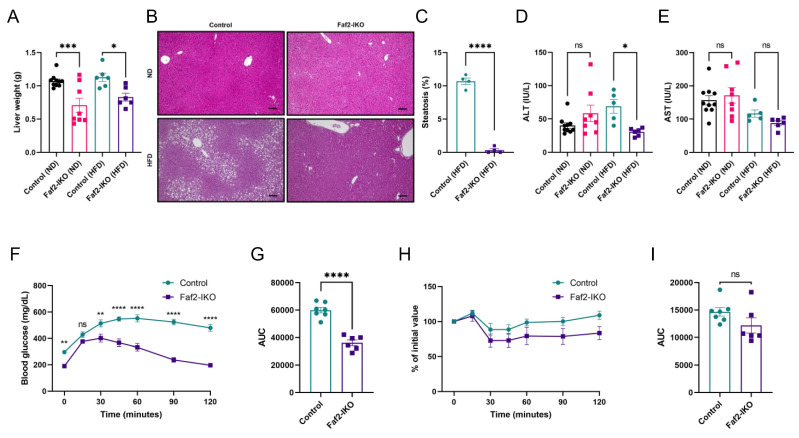
Intestine-specific Faf2 deficiency protects mice from HFD-induced HS and enhances glucose tolerance. 4-week-old male Faf2-IKO and littermate mice were fed a ND or HFD for 12 weeks. (A) Liver weight of ND-fed and HFD-fed male mice (n = 10 for WT ND, 8 for Faf2-IKO ND, 6 for WT HFD, 6 for Faf2-IKO HFD). (B) Images of H&E staining in the livers of male Faf2-IKO mice and Control mice fed ND and HFD. Hepatic steatosis was quantified by calculating the percentage of lipid droplet-positive area relative to the total parenchymal area (C) (n = 4 per group). Scale bar: 200 µm. (D, E) Serum ALT (D) and AST (E) levels in male Control and Faf2-IKO mice fed a ND or HFD (n = 10 for WT ND, 8 for Faf2-IKO ND, 5 for WT HFD, 6 for Faf2-IKO HFD). (F, G) Glucose tolerance tests were performed on fasted (6 hours) male Faf2-IKO mice and Control mice after 12 weeks of HFD. Blood glucose concentrations were monitored over a 2 hours period following glucose administration (F). Bar graphs represent the AUC for all time points (G) (n = 7 for WT HFD,6 for Faf2-IKO HFD). (H, I) Insulin tolerance tests were performed on fasted (4 hours) male Faf2-IKO mice and Control mice after 13 weeks of HFD. Blood glucose concentrations were measured over 2 hours after the insulin injection (H). Bar graphs represent the AUC for all time points (I) (n = 7 for WT HFD,6 for Faf2-IKO HFD). Data are shown as mean ± SEM. ns: not significant, *p<0.05, **p<0.01, ***p<0.001, ****p<0.0001. Statistical analyses were performed using one-way ANOVA followed by Bonferroni post-hoc testing for panels A, D, and E; unpaired two-tailed Student's t-tests for panels C, G, and I; and two-way ANOVA followed by Bonferroni post-hoc testing for panels F and H. Abbreviations: ALT, alanine aminotransferase; AST, aspartate aminotransferase; AUC, area under the curve; Faf2, fas-associated factor family member 2; Faf2-IKO, intestine-specific Faf2 knockout; H&E, hematoxylin and eosin; HS, hepatic steatosis; HFD, high-fat diet; ND, normal diet; WT, wild type.

Given the central role of the liver in coordinating systemic lipid metabolism, we examined whether intestinal Faf2 deficiency influences hepatic lipid homeostasis under ND and HFD conditions in a sex- and diet-dependent manner. Liver weight was reduced in male Faf2-IKO mice under both ND and HFD conditions, whereas in female Faf2-IKO mice, liver weight was reduced under ND conditions but remained unchanged under HFD conditions (Fig. 2A; Fig. S2A). Histological analysis revealed similar liver morphology between control and Faf2-IKO males under ND conditions, whereas hepatic lipid accumulation was lower in HFD-fed Faf2-IKO males than in controls. Quantitative assessment demonstrated a decrease in the LD area and the proportion of the steatotic area in liver sections from HFD-fed males (Fig. 2B, C). In contrast, no differences in HS were detected in female mice under either dietary condition (Fig. S2B). Serum alanine transaminase (ALT) and aspartate aminotransferase (AST) levels were measured to assess liver injury. In male mice, ALT and AST levels were unchanged under ND. Under HFD, ALT levels were significantly reduced in Faf2-IKO mice, whereas AST levels were not significantly different between groups (Fig. 2D, E). In female mice, ALT and AST levels did not differ between genotypes under either ND or HFD conditions (Fig. S2C, D). Taken together, these findings indicate that intestine-specific Faf2 deletion is associated with reduced hepatic lipid accumulation and lower serum ALT levels in HFD-fed male mice, whereas hepatic histology and injury markers remained unchanged in females.

Next, we evaluated whether Faf2 deficiency affected glucose metabolism. Following glucose administration, Faf2-IKO mice of both sexes on an HFD demonstrated improved glucose tolerance (Fig. 2F, G; Fig. S2E, F); however, no significant differences were observed in blood glucose levels during insulin tolerance testing compared to littermates (Fig. 2H, I; Fig. S2G, H). These findings suggest that Faf2 deficiency improves glucose tolerance under HFD conditions without altering systemic insulin sensitivity.

### 3.3. Intestinal Faf2 deficiency does not change RER but is associated with altered energy absorption

**Figure 3:**
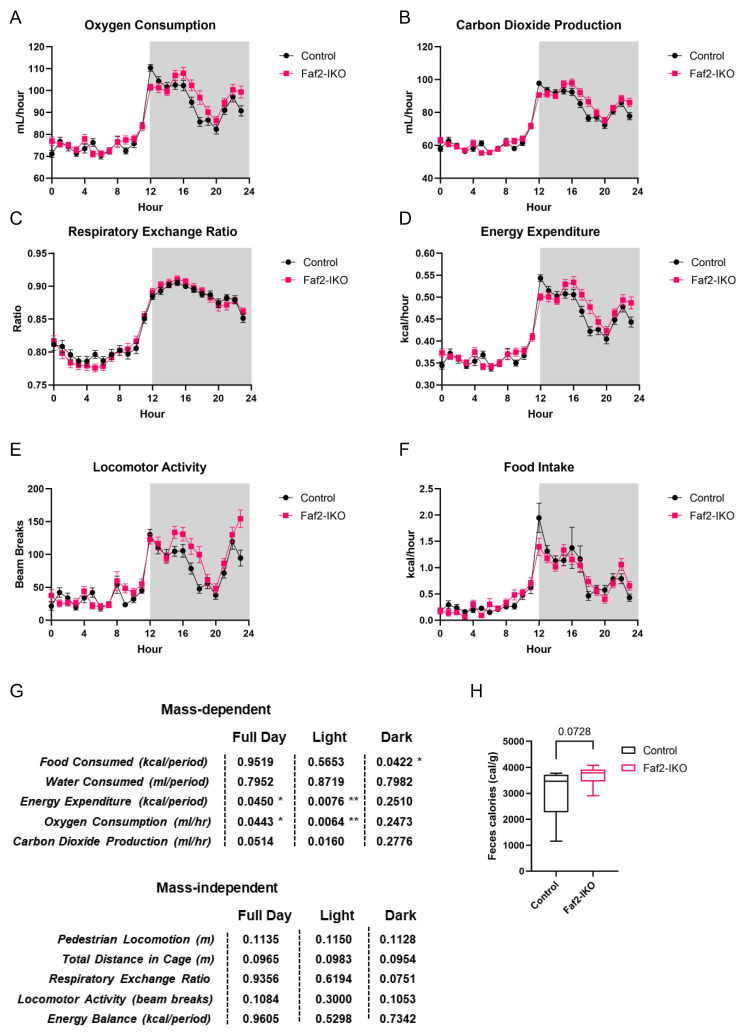
Metabolic profile of Faf2-IKO male mice. (A) Analyses of oxygen consumption, (B) carbon dioxide production, (C) respiratory exchange ratio, (D) energy expenditure, (E) locomotor activity, and (F) food intake were conducted in 12-week-old Faf2-IKO male mice and littermates fed a ND (n = 7 per group). (G) Summary of ANCOVA and ANOVA statistical analyses for each metabolic parameter. (H) Fecal bomb calorimetry analysis of the feces of 12-week-old Faf2-IKO male mice and littermates fed ND (n = 7 per group). Data are shown as mean ± SEM. ns: not significant, *p<0.05, **p<0.01. Statistical analyses were performed using two-way ANOVA for C, E, ANCOVA for A, B, D, F, and unpaired two-tailed Student's t-tests for H. Abbreviations: Faf2, fas-associated factor family member 2; Faf2-IKO, intestine-specific Faf2 knockout; ND, normal diet.

Since intestinal Faf2 deletion resulted in both reduced adiposity and improved hepatic lipid profiles, we investigated whether these effects were linked to alterations in systemic energy balance. Faf2-IKO males showed slightly higher EE and VO₂ during the full day and light photoperiods. However, there were no differences in the RER, locomotor activity, food intake, or energy balance (Fig. 3A-G). Additionally, Faf2-IKO males showed a trend toward increased energy content in the feces (Fig. 3H), which may be consistent with altered energy absorption. Faf2-IKO females displayed decreased EE, VO₂, VCO₂, and water consumption over 24 hours (most evident during the light phase). Although the pattern in females differs from that in males, this difference may reflect variation in body mass. Even within a single species, comparisons are constrained by the extent to which mass and composition diverge. Notably, Faf2-IKO females were lighter than controls in this study. Importantly, the RER and food intake were not different, locomotor activity differed primarily in the dark phase, 24-hour energy balance remained unchanged (Fig. S3A-G), and fecal caloric content was significantly higher in Faf2-IKO females (Fig. S3H), which may similarly reflect altered energy absorption. Overall, Faf2-IKO males showed slightly elevated EE/VO₂, whereas Faf2-IKO females showed lower values for these variables. Despite this divergence, female Faf2-IKO mice exhibited significantly higher fecal energy content, while male Faf2-IKO mice showed a similar tendency, potentially consistent with altered energy absorption efficiency.

### 3.4. Intestine-specific Faf2 depletion alters circulating lipid and lipoprotein profiles

**Figure 4:**
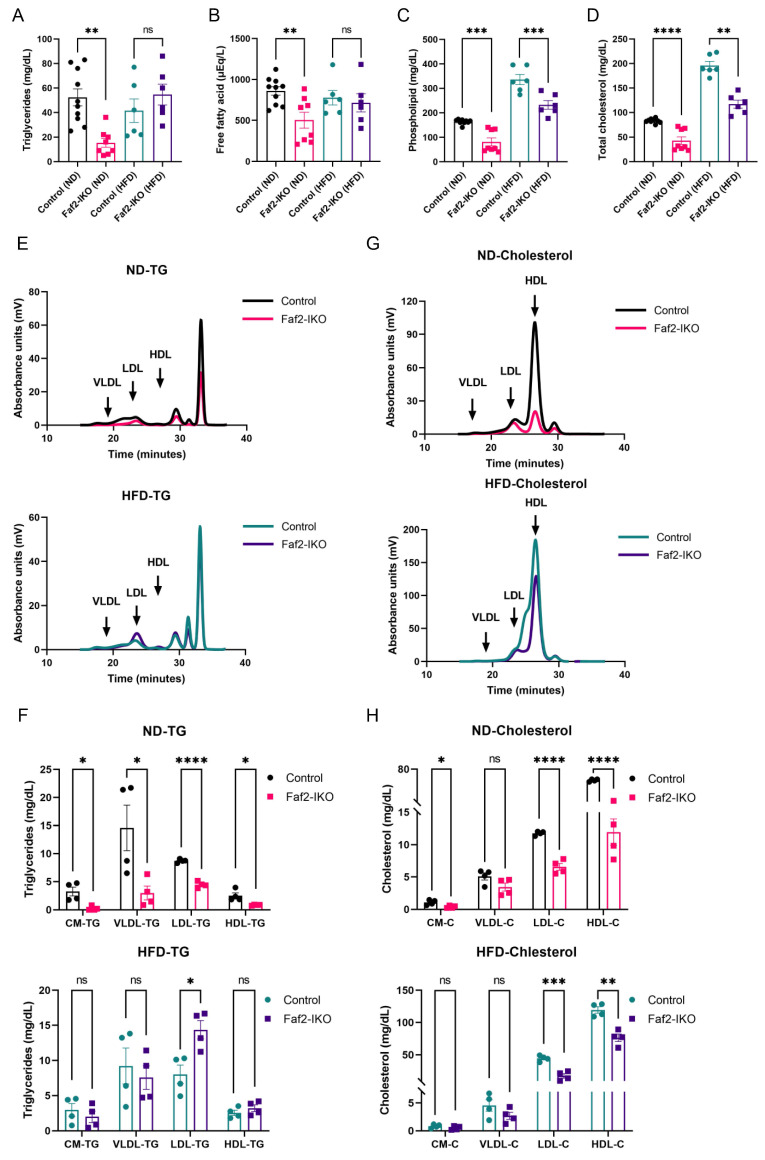
Faf2 is essential for efficient intestinal lipid metabolism. (A-D) Plasma levels of TG (A), FFA (B), phospholipids (C), and total cholesterol (D) were measured in male mice fed ND or HFD (n = 10 for WT ND, 8 for Faf2-IKO ND,6 for WT HFD,6 for Faf2-IKO HFD). (E, G) Lipoprotein TG (E) and cholesterol (G) profiles of Control male mice and Faf2-IKO mice fed either ND or HFD (n = 4 per group), as determined by GP-HPLC analysis. (F, H) TG (F) and cholesterol (H) concentrations were measured in each lipoprotein fraction from Control male mice and Faf2-IKO mice fed either ND or HFD (n = 4 per group). Data are shown as mean ± SEM. ns: not significant, *p < 0.05, **p < 0.01, ***p < 0.001, ****p < 0.0001. Statistical analyses were performed using one-way ANOVA followed by Bonferroni post-hoc testing for panels A-D, and multiple unpaired two-tailed Student's t-tests without correction for multiple comparisons for panels F and H. Abbreviations: CM, chylomicron; C, cholesterol; Faf2, fas-associated factor family member 2; Faf2-IKO, intestine-specific Faf2 knockout; FFA, free fatty acid; GP-HPLC, gel permeation high-performance liquid chromatography; HDL, high-density lipoproteins; HFD, high-fat diet; LDL, low-density lipoproteins; ND, normal diet; TG, triglycerides; VLDL, very low-density lipoprotein; WT, wild type.

To investigate whether intestinal changes affect systemic lipid homeostasis, we analyzed the blood lipid parameters under different dietary conditions. Faf2-IKO male mice showed reduced plasma lipid levels under ND conditions, with phospholipids and cholesterol remaining significantly decreased, even under HFD conditions (Fig. 4A-D). Similarly, the levels of several lipid species were reduced in female HFD-fed Faf2-IKO mice (Fig. S4A-D), suggesting that intestinal Faf2 deficiency influences systemic lipid metabolism across both sexes. Next, to quantify the TG and cholesterol contents across the lipoprotein subclasses, we employed gel filtration HPLC 21. Notably, the TG content in the CM, VLDL, low-density lipoprotein (LDL), and HDL subclasses of blood samples from Faf2-IKO male mice fed a ND was decreased, but there were no differences in Faf2-IKO male mice fed an HFD, with the exception of the LDL subclass, which remained significantly altered (Fig. 4E, F). In addition, the cholesterol content in the CM, LDL, and HDL subclasses of blood samples from Faf2-IKO male mice fed a ND was reduced. In Faf2-IKO male mice fed an HFD, decreases in cholesterol content were also observed in the LDL and HDL subclasses (Fig. 4G, H). These findings suggest that intestinal Faf2 deficiency is associated with altered circulating lipid and lipoprotein profiles, particularly under ND conditions, whereas the effect appears to be partially attenuated in the presence of excess dietary lipid intake.

### 3.5. Intestine-specific Faf2 depletion reduces CM formation

**Figure 5:**
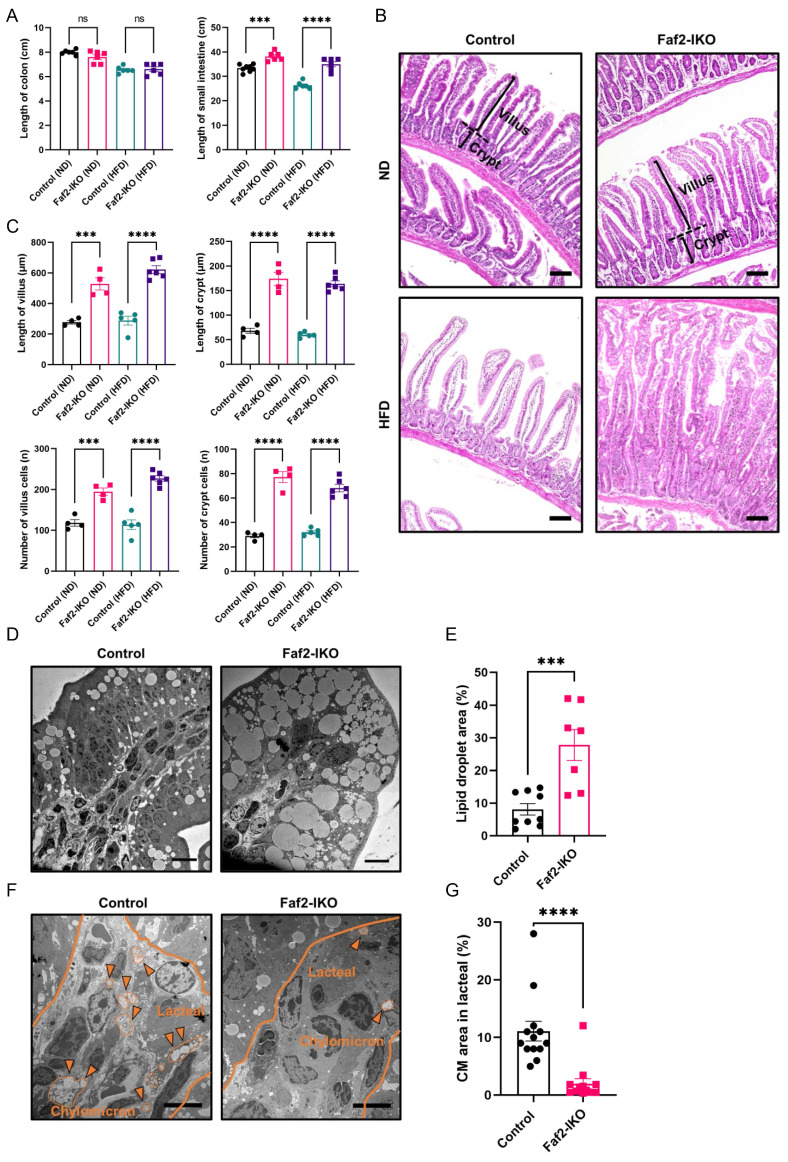
Intestinal Faf2 deficiency alters intestinal lipid handling and reduces CM abundance in the small intestine. (A) The lengths of the colon and small intestine were measured in male mice fed an ND or HFD. Small intestine length was defined as the distance from the ligament of Treitz to the cecum (n = 6, 8 for WT ND, 6 for Faf2-IKO ND,6 for WT HFD,6 for Faf2-IKO HFD). (B) From H&E-stained slides of jejunum, crypt-villus junctions were established (dotted line) and villus height and crypt depth (brackets). Scale bars: 100 µm (C) Villus length and crypt length were measured using Image J software, while villus and crypt cell numbers were determined by counting the visible nuclei in the epithelial layer in mice fed a ND or HFD (n > 48 crypts or n > 20 villi from 4-6 mice per group). (D) Representative transmission electron micrographs of jejunal enterocytes from ND-fed male Control and Faf2-IKO mice fasted for 16 h and analyzed 2 h after oral gavage of olive oil (10 μL/g body weight). Images were acquired at magnifications of ×8,000-×30,000. For each mouse, at least 10 non-overlapping fields from two independent ultrathin sections were analyzed. Scale bar: 20 µm. (E) Quantitative analyses of LD area in jejunal epithelial cells (n = 486 from 3 mice for WT ND; n = 1877 from 3 mice for Faf2-IKO ND). (F, G) Representative electron micrographs (F) and quantification (G) of CM particles in jejunal lacteals from ND-fed male Control and Faf2-IKO mice under the same experimental conditions as in (D). Fields containing intact lacteals were randomly selected, and analyses were performed in a blinded manner. The number of CM particles in each lacteal field was determined (n = 1183 from 3 mice for WT ND; n = 233 from 3 mice for Faf2-IKO ND). The outlined region denotes the villus lacteal; circled particles are CM, indicated by arrows. Scale bars: 5 µm. Data are shown as mean ± SEM. ns: not significant, ***p<0.001, ****p<0.0001. Statistical analyses were performed using one-way ANOVA followed by Bonferroni post-hoc testing for panels A and C, and unpaired two-tailed Student's t-tests for panels E and G. Abbreviations: CM, chylomicrons; Faf2, fas-associated factor family member 2; Faf2-IKO, intestine-specific Faf2 knockout; H&E, hematoxylin and eosin; HFD, high-fat diet; LD, lipid droplets; ND, normal diet; WT, wild type.

Next, we assessed whether structural remodeling of the intestine was associated with altered nutrient absorption in Faf2-IKO mice. Although colon length remained unchanged, male and female Faf2-IKO mice exhibited increased small intestine length (defined as the distance from the ligament of Treitz to the cecum) (Fig. 5A; Fig. S6A) and showed elongated villi and crypts with increased villus and crypt cell counts (Fig. 5B, C; Fig. S6B, C) under ND and HFD conditions, respectively. In contrast, no differences in the small intestine length or villus and crypt cell counts were detected between control and Faf2-IKO male mice at 4 weeks of age under ND conditions (Fig. S5A, B), suggesting that these intestinal structural differences emerge after weaning rather than reflecting an overt developmental alteration. Together, these findings suggest that intestine-specific Faf2 deletion is associated with post-weaning expansion of the intestinal absorptive surface rather than a primary developmental defect. To assess epithelial cell proliferation during post-weaning intestinal expansion, Ki67 immunohistochemistry was performed. No differences in Ki67-positive epithelial cells were observed between the control and Faf2-IKO male and female mice under ND conditions (Fig. S5C, D; Fig. S6D, E), suggesting that the increased villus and crypt cell counts are not explained by enhanced steady-state epithelial proliferation at the time examined. Instead, these findings may reflect alternative mechanisms involved in post-weaning intestinal expansion, including reduced epithelial cell loss, altered epithelial migration along the crypt-villus axis, or post-weaning intestinal remodeling processes such as crypt fission 25,26.

To determine how these structural changes affect lipid handling, an ultrastructural analysis of enterocytes was then performed. In ND-fed male Faf2-IKO mice, enterocytes exhibited increased LD accumulation, which were enlarged and occupied a greater proportion of the cytoplasm than controls (Fig. 5D). Quantitative analysis confirmed an increase in the LD area (Fig. 5E), consistent with impaired handling of absorbed lipids. In contrast, the jejunal lacteals of Faf2-IKO male mice contained fewer CMs. Although lacteals from control mice were densely filled with CMs, those from male Faf2-IKO mice contained relatively sparse particles, resulting in a reduction in the CM area (Fig. 5F, G). Collectively, these findings indicate increased intracellular lipid retention in enterocytes and reduced CM presence in lacteals in the absence of intestinal Faf2, indicating an altered transfer of dietary lipids from enterocytes to lymphatic circulation.

### 3.6. Intestinal Faf2 deficiency is associated with ApoB accumulation, ER alterations, and altered enterocyte lipid handling

**Figure 6:**
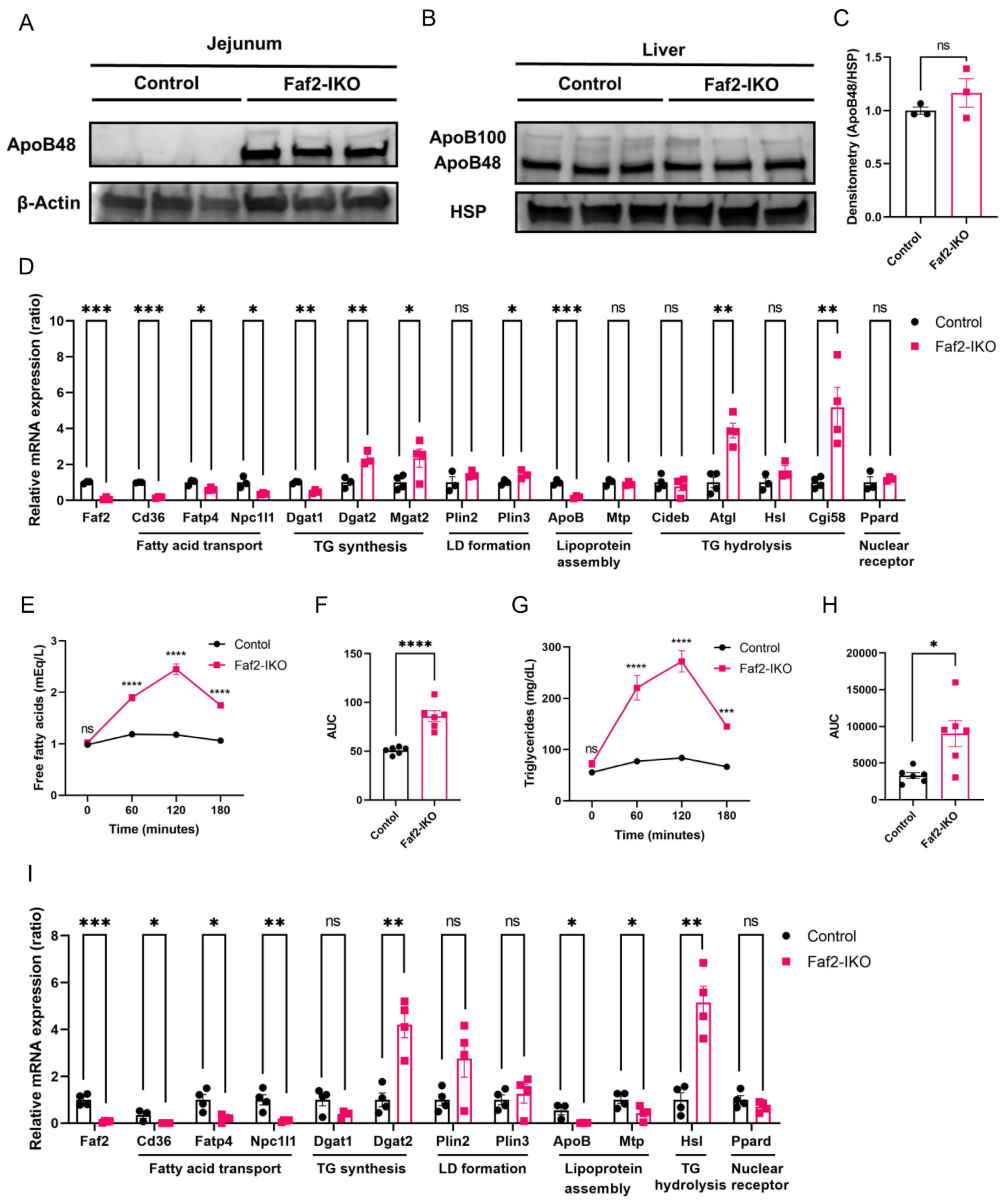
Intestinal Faf2 deficiency leads to ApoB protein accumulation, altered enterocyte lipid handling, and abnormal postprandial lipid responses. (A) Representative immunoblots showing ApoB48 protein levels in the jejunum of ND-fed male Control and Faf2-IKO mice. β-Actin was used as a loading control. (B, C) Representative immunoblots of hepatic ApoB100 and ApoB48 protein levels in ND-fed male Control and Faf2-IKO mice (B). HSP was used as a loading control. Densitometric quantification of hepatic ApoB48 protein levels normalized to HSP in (C) (n = 3 per group). (D) Quantitative PCR analysis of mRNA-related lipid metabolism levels in jejunum of ND-fed male Control and Faf2-IKO male mice (n = 3 or 4 per group). Cyclophilin A was used as the internal control. (E, F) Plasma FFA levels (E) and AUC (F) in ND-fed Control and Faf2-IKO male mice following a LTT with olive oil gavage (10 μL/g body weight) after 16 h fasting (n = 6 per group). (G, H) Plasma TG levels (G) and AUC (H) in the same experimental setting in Faf2-IKO mice (n = 6 per group). (I) Quantitative PCR analysis of mRNA-related lipid metabolism levels in jejunum of ND-fed male Control and Faf2-IKO male mice after LTT (n = 3 or 4 per group). Cyclophilin A was used as the internal control. Data are shown as mean ± SEM. ns: not significant, *p<0.05, **p<0.01, ***p<0.001, ****p<0.0001. Statistical analyses were performed using unpaired two-tailed Student's t-tests for panels C, F, and H; multiple unpaired two-tailed Student's t-tests without correction for multiple comparisons for panels D and I; and two-way ANOVA followed by Bonferroni post-hoc testing for panels E and G. Abbreviations: ApoB, apolipoprotein B; AUC, area under the curve; Faf2, fas-associated factor family member 2; Faf2-IKO, intestine-specific Faf2 knockout; FFA, free fatty acids; HSP, heat shock protein; LD, lipid droplet; LTT, lipid tolerance test; mRNA, messenger RNA; TG, triglycerides.

Because ApoB48 is an essential structural component for intestinal CM formation and secretion, we examined ApoB expression in the intestine 8. In ND-fed Faf2-IKO male mice, ApoB protein levels were elevated in the jejunum relative to controls (Fig. 6A), whereas hepatic ApoB levels were unchanged (Fig. 6B, C). These findings indicate the tissue-specific accumulation of ApoB in enterocytes, suggesting that ApoB-containing lipoproteins are synthesized but are not efficiently secreted into lacteals.

Given that ApoB synthesis and lipidation are closely associated with ER function, we next examined whether ER morphology and ER-related responses were altered in Faf2-deficient enterocytes. Transmission electron microscopy revealed dilation of the rough ER in enterocytes from Faf2-IKO mice compared with controls (Fig. S7A, B). Consistent with these ultrastructural changes, jejunal qPCR analysis showed increased expression of genes related to ER homeostasis and vesicle trafficking, including *Sar1b* and *Hspa5* (Fig. S7C), which may reflect altered ER homeostasis in Faf2-deficient enterocytes.

To further investigate molecular pathways potentially associated with the altered enterocyte lipid phenotype observed in Faf2-IKO mice, we next examined the expression of genes involved in intestinal lipid uptake and CM assembly. Quantitative PCR analysis of jejunal samples further revealed transcriptional changes in Faf2-IKO mice compared with controls. Specifically, the expression levels of genes involved in intestinal lipid uptake and CM assembly, such as *Cd36*, *Fatp4*, *Npc1l1*, *ApoB*, and *Mtp*, were downregulated in the jejunum of Faf2-IKO mice, which may be consistent with reduced lipid uptake and export capacity. In contrast, genes that promote TG synthesis and LD storage (*Dgat2* and *Plin3*), as well as lipolysis enzymes (*Hsl* and *Atgl*) were elevated in the Faf2-IKO jejunum (Fig. 6D, Fig. S8A). Collectively, these transcriptional changes are consistent with altered intracellular lipid handling and increased lipid retention within enterocytes.

To determine whether impaired intestinal lipid export translated into altered postprandial lipid handling, an LTT was performed. Following oral lipid loading, control mice showed a gradual increase in plasma FFA and TG levels, whereas Faf2-IKO mice exhibited higher postprandial levels with delayed normalization, resulting in increased AUCs for both FFAs and TGs (Fig. 6E-H; Fig. S9A-D). Despite reduced CM abundance in lacteals, this paradoxical pattern suggested altered systemic regulation of postprandial lipid metabolism in Faf2-IKO mice.

To further assess whether intestinal transcriptional changes alone could account for these postprandial responses, jejunal gene expression was analyzed following the LTT. Compared with controls, the Faf2-IKO jejunum showed reduced expression of genes related to lipid-uptake/transport and CM-assembly transcripts, with concomitant induction of TG synthesis, LD, and lipolysis modules (Fig. 6I; Fig. S8B). Overall, these transcriptional changes did not readily explain the elevated circulating TG and FFA levels observed following oral fat loading. This apparent discrepancy represents an unresolved aspect of the current study. Because postprandial circulating lipid levels are regulated by multiple systemic metabolic processes beyond the intestine, the elevated plasma TG and FFA levels observed in Faf2-IKO mice could not be readily explained by the present intestinal transcriptional findings alone. Although extra-intestinal processes such as adipose tissue lipolysis and hepatic lipoprotein production may potentially contribute, their involvement remains speculative because these parameters were not directly assessed under LTT conditions. To further explore systemic transcriptional changes associated with intestinal Faf2 deficiency, hepatic RNA-seq analysis was performed.

### 3.7. Identification of DEGs in the livers of Faf2-IKO mice fed an HFD

**Figure 7:**
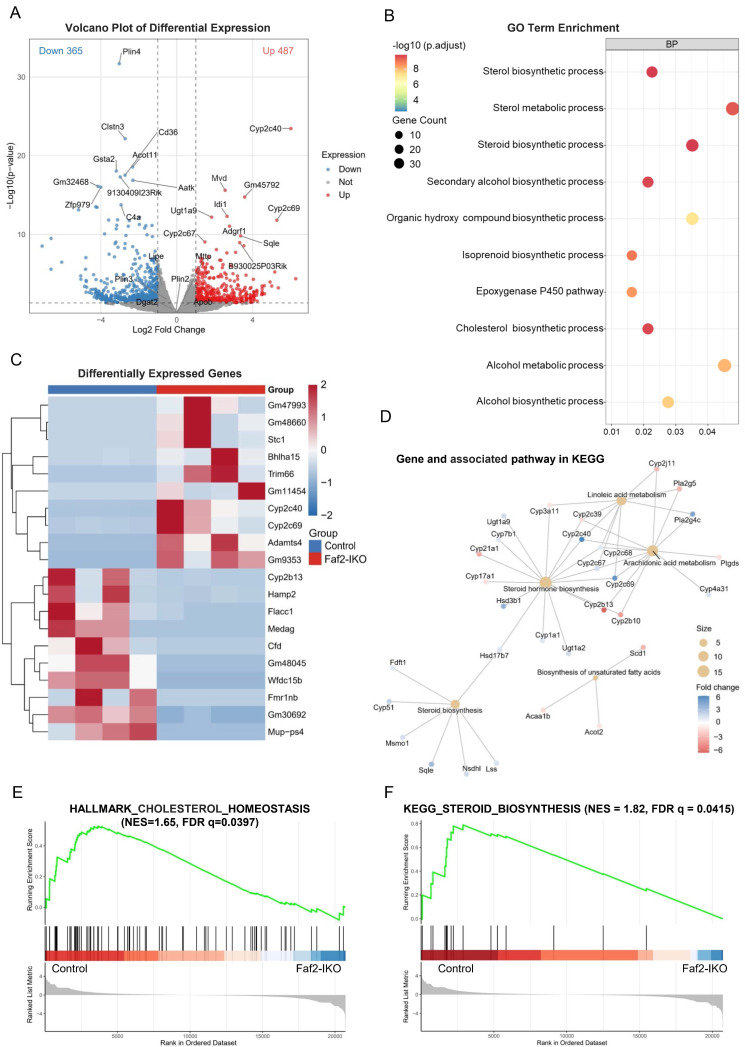
Intestinal Faf2 deficiency alters hepatic gene expression associated with cholesterol and FA metabolism. (A) Volcano plot showing DEGs in the livers of HFD-fed male Faf2-IKO mice versus Control mice. Red and blue dots represent significantly upregulated and downregulated genes, respectively (|log₂ fold change| > 2, adjusted p < 0.05). Notable genes involved in lipid metabolism are labeled. (B) GO enrichment analysis (BP) of DEGs revealed significant enrichment in sterol biosynthesis, steroid biosynthesis, and metabolic processes. Dot size reflects gene count; color indicates statistical significance (adjusted p-value). (C) Heatmap showing the hierarchical clustering of selected DEGs in the liver between Faf2-IKO and Control groups. Log₂-transformed expression values are color-coded, with red indicating upregulation and blue indicating downregulation. (D) KEGG pathway network analysis of lipid metabolic genes highlights the pathways affected by Faf2 deletion, including steroid hormone biosynthesis, arachidonic acid metabolism, and unsaturated FAs. Node size reflects gene number, and color represents log₂ fold change. (E) GSEA of the HALLMARK_CHOLESTEROL_HOMEOSTASIS pathway demonstrated positive enrichment in Faf2-IKO mice. (F) GSEA of the KEGG_ STEROID_BIOSYNTHESIS pathway shows positive enrichment in Faf2-IKO male mice (n = 4 per group). Abbreviations: BP, biological processes; DEGs, Differentially Expressed Genes; FA, fatty acid; Faf2, fas-associated factor family member 2; Faf2-IKO, intestine-specific Faf2 knockout; FDR, false discovery rate; GO, Gene Ontology; GSEA, Gene Set Enrichment Analysis; KEGG, Kyoto Encyclopedia of Genes and Genomes; NES, normalized enrichment scores.

Given the central role of the liver in lipid handling, genome-wide transcriptomic profiling is well-suited for revealing transcriptional changes associated with this response. In Faf2-IKO livers, 487 genes were upregulated, and 365 were downregulated (Fig. 7A), indicating broad transcriptional remodeling. Gene Ontology analysis highlighted lipid-anabolic programs, most prominently sterol/steroid/cholesterol biosynthetic processes and P450-mediated lipid metabolism (Fig. 7B). A representative heat map shows the coordinated induction of the lipid-handling modules (Fig. 7C). KEGG pathway analysis revealed that network-level enrichment focused on steroid/sterol biosynthesis and unsaturated FA biosynthesis, with sequential mevalonate/sterol enzymes (e.g., *Sqle*, *Lss*, *Msmo1*, *Nsdhl*, *Hsd17b7*) coordinately upregulated, consistent with pathway-level transcriptional enrichment rather than isolated single-gene changes (Fig. 7D). Concordantly, rank-based GSEA demonstrated significant positive enrichment of cholesterol homeostasis and steroid biosynthesis pathways in Faf2-IKO livers (Fig. 7E, F).

These findings suggest that intestinal Faf2 deficiency is associated with hepatic transcriptional enrichment of sterol- and lipid biosynthetic pathways. Although these transcriptional changes may be consistent with altered systemic lipid metabolic states, the underlying physiological significance remains unclear. Importantly, hepatic VLDL secretion, *de novo* lipogenesis, and sterol synthesis flux were not directly measured in the present study. Therefore, the hepatic RNA-seq results should primarily be interpreted as transcriptional alterations associated with intestinal Faf2 deficiency rather than direct evidence of altered hepatic lipid metabolic flux. In contrast, skeletal muscle RNA-seq identified 421 downregulated and 482 upregulated genes, the DEG heatmap and GO enrichment were dominated by muscle system process/contractile and cytoskeletal terms, and antigen processing/presentation, with no significant enrichment of canonical lipid-handling pathways (Fig. S10A-C). Together, these findings suggest coordinated alterations in intestinal lipid handling and hepatic transcriptional programs related to sterol- and lipid-associated pathways in Faf2-IKO mice.

## 4. Discussion

**Figure 8:**
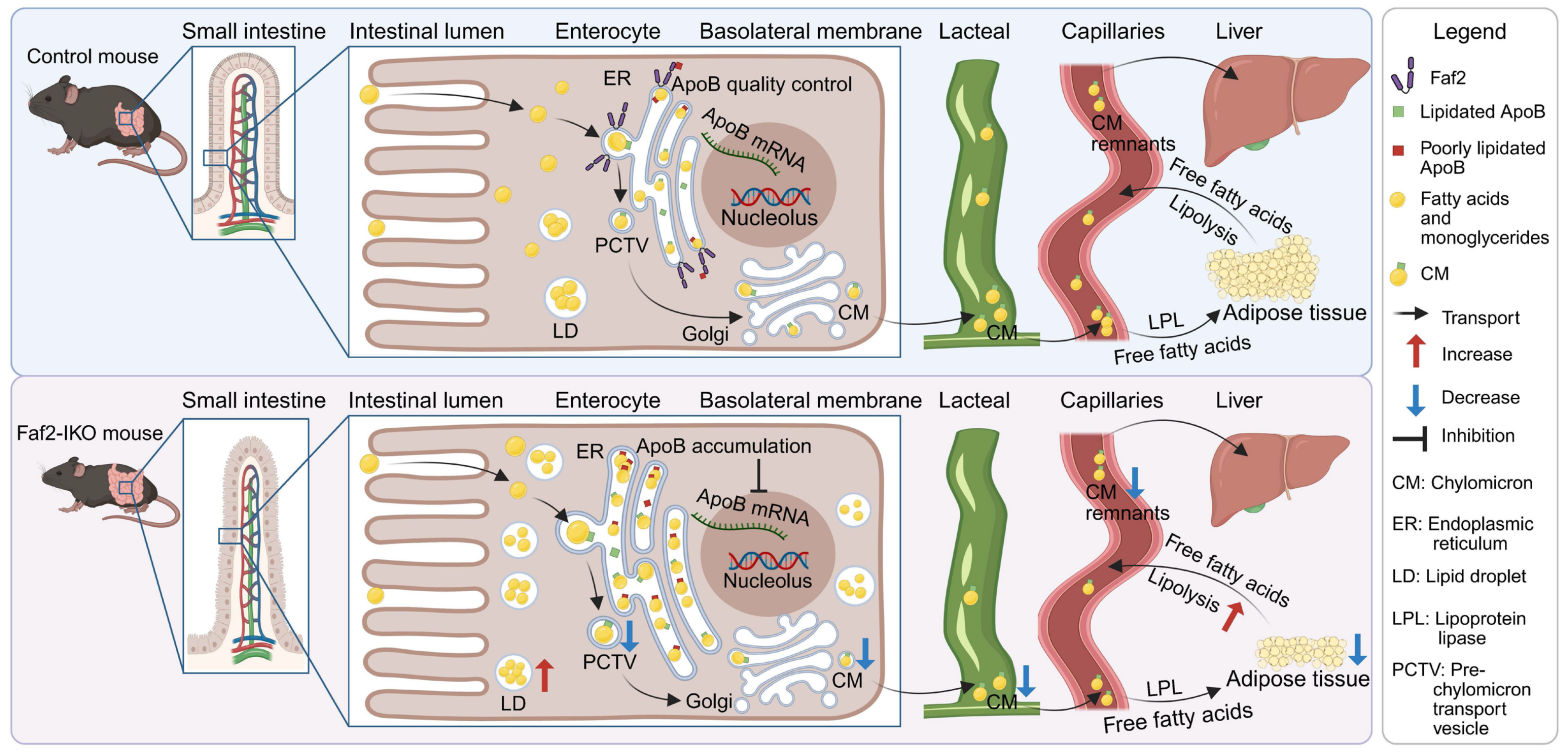
Proposed model of the metabolic alterations associated with intestinal Faf2 deficiency. Dietary lipids are absorbed into enterocytes, where ApoB48-containing lipoproteins are assembled within the ER, transported through the Golgi apparatus, and secreted as CMs into intestinal lacteals for systemic lipid distribution to adipose tissue and the liver. Intestine-specific deletion of Faf2 was associated with increased intracellular ApoB protein accumulation in enterocytes, accompanied by enhanced LD retention, dilation of the rough ER. These alterations were accompanied by reduced CM abundance in intestinal lacteals, consistent with altered intestinal lipid and lipoprotein handling. Intestinal Faf2 deficiency was also associated with reduced adipose tissue mass and attenuated hepatic lipid accumulation. In addition, increased adipose expression of lipid mobilization-related genes may be consistent with altered systemic lipid metabolism in Faf2-IKO mice. Collectively, these findings support a potential relationship between intestinal Faf2 deficiency, altered enterocyte lipid handling, and systemic metabolic phenotypes in mice. Created in BioRender. ZHANG, J. (2026) https://BioRender.com/2x0d7v5. Abbreviations: ApoB, apolipoprotein B; CM, chylomicron; ER, endoplasmic reticulum; Faf2, Fas-associated factor family member 2; Faf2-IKO, intestine-specific Faf2 knockout; LD, lipid droplet; LPL, lipolysis; mRNA, messenger RNA; PCTV, pre-chylomicron transport vesicle; TG, triglyceride.

This study supports a role for intestinal Faf2 in the regulation of lipid absorption and the pathogenesis of HFD-induced HS and obesity in mice. The intestine-specific deletion of Faf2 resulted in increased ApoB protein retention in enterocytes, accompanied by enhanced LD accumulation, reduced CM abundance in lacteals, together with altered postprandial lipid handling, thereby attenuating HFD-induced steatosis and adiposity and improving glucose tolerance (Fig. 8). These alterations were associated with structural and molecular changes related to ER homeostasis, including ER dilation and altered expression of genes associated with ER homeostasis, suggesting involvement of altered ER-associated lipid handling processes in Faf2-deficient enterocytes. Collectively, our findings support a role for intestinal Faf2 in ER-associated ApoB-containing lipoprotein handling and intestinal lipid transport, although the precise molecular mechanisms linking Faf2 deficiency to altered ER homeostasis, ApoB retention, and impaired CM output remain to be further elucidated.

Genome-wide association and meta-analytic data have linked polymorphisms in Faf2 to a lower risk of alcohol-related cirrhosis in humans 27 and recent animal studies suggest that reducing Faf2 activity mitigates alcohol-induced steatosis by dampening SREBP1 signaling and enhancing ATGL-mediated lipolysis 28. Our previous study showed that the hepatocyte-specific loss of Faf2 impairs ApoB proteasomal degradation, thereby suppressing VLDL secretion 2. However, the physiological role of Faf2 in the intestine has remained largely unclear. Because the small intestine depends on ApoB48-mediated CM formation for dietary lipid export, we investigated whether intestinal Faf2 deficiency influences intestinal lipid handling and intestine-liver metabolic communication. In the present study, intestinal Faf2 deficiency was associated with increased ApoB protein retention in enterocytes, reduced CM abundance in lacteals, and marked intracellular LD accumulation, collectively suggesting altered intestinal lipoprotein handling and CM export. Although ApoB mRNA expression was reduced in the jejunum of Faf2-IKO mice, this finding should be interpreted with caution and may reflect broader alterations in enterocyte lipid metabolic states rather than direct evidence defining the precise mechanism underlying ApoB48 handling.

Recent advances have refined our understanding of how ApoB trafficking and turnover constrain lipoprotein output in both the liver and intestine. ApoB100 in the liver assembles into VLDL, whereas the small intestine relies on ApoB48 to generate CM 9. MTTP is essential for ApoB lipidation and CM/VLDL production, and its loss abolishes lipoprotein output and leads to severe enterocyte lipid accumulation and mucosal pathology 29-31. In contrast to this binary “on-off” gate, our data indicate that intestinal Faf2 deficiency is associated with altered intestinal lipoprotein handling and reduced CM abundance, without the severe mucosal pathology characteristic of MTTP blockade. Importantly, the current data do not distinguish among potential underlying mechanisms, which may include altered ApoB processing, impaired ER-to-Golgi trafficking, or changes in MTTP-dependent lipidation. These distinctions underscore differences in biological magnitude and pathological consequences between MTTP inhibition and intestinal Faf2 deficiency and raise the possibility that partial modulation of intestinal CM assembly may influence hepatic lipid homeostasis while potentially avoiding some of the safety concerns associated with global MTTP inhibition.

A previous study reported that in C57BL/6J mice, a BW of 41.6 g marks the threshold at which hepatic TG accumulation is accelerated 32. In our study, all HFD-fed males remained below 41.6 g. Particularly, Faf2-IKO mice averaged 23 g, making post-HFD weight matching infeasible. Importantly, food intake did not differ between control and Faf2-IKO mice in either sex (Fig. 3F; Fig. S3F), indicating that the reduced BW in Faf2-IKO mice is more consistent with impaired intestinal lipid absorption rather than reduced food intake.

Notably, Faf2-IKO mice exhibited increased small intestinal length and villus expansion after weaning, but showed no evidence of increased steady-state epithelial proliferation at the time examined, as assessed by Ki67 staining. This apparent discrepancy suggests that mechanisms beyond enhanced proliferation may contribute to intestinal remodeling. Possible explanations include reduced epithelial cell loss, altered migration dynamics along the crypt-villus axis, or post-weaning intestinal remodeling processes, including crypt fission. These processes are well recognized as key determinants of intestinal architecture and epithelial homeostasis, operating independently of steady-state proliferation rates 25,26,33. Although these mechanisms were not directly examined in the present study, they provide a plausible biological framework for interpreting the observed post-weaning intestinal expansion.

In intestinal Faf2-IKO mice, several observations converged on the sex-specific biology. First, metabolic-cage readouts revealed sex-dimorphic energy balance, Faf2-IKO males showed EE/VO₂ increases, whereas females showed reductions in these parameters. Second, intestinal Faf2 deletion lowered liver weight and HS in males, whereas females remained relatively lean and barely showed HS, even after 12 weeks of HFD feeding (Fig. S2A, B). Notably, previous hepatocyte-specific Faf2 knockout studies have reported a stronger hepatic phenotype in females, including periportal macrovesicular steatosis and broader abnormalities 2. These contrasting phenotypes may reflect differences in sex-dependent metabolic regulation. On the hepatic side, estrogen signaling through ERα, partly via ERRα-dependent transcriptional programs, supports mitochondrial oxidative metabolism and promotes efficient lipid utilization, thereby limiting ectopic lipid accumulation under obesogenic conditions. In addition, ERα/ERRα signaling has been shown to facilitate hepatic VLDL assembly and secretion, providing a buffering mechanism for excess lipid export and contributing to sex differences in NAFLD/NASH susceptibility 34,35. Accordingly, it is possible that the stronger hepatic phenotype previously observed in female hepatocyte-specific Faf2 knockout mice may reflect differences in the ability to adapt to altered ApoB-containing lipoprotein metabolism 2. On the intestinal side, in Faf2-IKO males, CM secretion is reduced, which may limit the delivery of dietary lipids to the liver. The observed increase in EE/VO₂ in males may reflect an adaptive response to altered nutrient handling, potentially contributing to reduced hepatic lipid burden. In females, estrogen-associated metabolic features may limit hepatic lipid accumulation under HFD conditions by promoting more efficient lipid oxidation. This pre-existing metabolic profile may attenuate the impact of reduced intestinal lipid delivery, thereby rendering additional changes in whole-body energy expenditure or hepatic lipid accumulation less apparent following intestinal Faf2 deletion 36-38.

Liver RNA-seq revealed enrichment of sterol/steroid biosynthesis, fatty acid metabolism, and cholesterol homeostasis pathways, accompanied by coordinated downregulation of Cd36 and Acot11. These transcriptional changes may reflect adaptive hepatic remodeling associated with altered intestinal lipid delivery in Faf2-IKO mice. Reduced hepatic Cd36 expression, a transporter involved in long-chain fatty-acid uptake and previously associated with hepatic steatosis 39,40, may be compatible with altered hepatic fatty-acid handling under conditions of impaired intestinal lipid export. Similarly, reduced Acot11 expression may indicate altered acyl-CoA metabolism and lipid metabolic adaptation 41. In skeletal muscle, GO terms demonstrated enrichment of muscle system process and actin binding pathways rather than lipid-metabolic pathways; moreover, the SM-to-BW ratio was unchanged between genotypes (Fig. 1H), supporting the idea that the major metabolic alterations associated with intestinal Faf2 deficiency are more prominently linked to the intestine-liver-adipose axis than to skeletal muscle lipid metabolism.

### Limitations and future directions

This study has some limitations. First, despite the increased ApoB48 protein accumulation in enterocytes and reduced CM abundance in lacteals, we did not directly quantify CM secretion rates or determine the specific intracellular step(s) involved in ApoB-containing lipoprotein trafficking and export. Although ER dilation together with altered expression of genes related to ER-associated cellular responses supports the involvement of altered ER homeostasis in Faf2-deficient enterocytes, the precise stage at which ApoB processing is disrupted remains unresolved. Further mechanistic studies investigating ApoB48 trafficking and ER-associated lipid handling processes will be important to clarify the intracellular defects associated with intestinal Faf2 deficiency 42-44.

Second, the paradoxical elevation of circulating FFA and TG levels during the LTT, despite evidence of impaired intestinal CM secretion, remains unresolved. The precise sources of these elevated postprandial lipids could not be determined because direct functional analyses of intestinal lipid flux, adipose lipolysis during the LTT, and hepatic VLDL secretion were not performed. Although lipolysis-related genes were increased in the iWAT of ND-fed Faf2-IKO mice, these observations were obtained under basal ND conditions and therefore cannot directly account for the postprandial FFA/TG excursions. Future studies incorporating lipid tracing approaches, hepatic VLDL secretion analyses, hormone profiling such as incretin responses 45,46 and stable isotope flux measurements 47 will be required to clarify the mechanisms underlying this paradoxical postprandial lipid phenotype.

Third, although hepatic RNA-seq identified coordinated transcriptional changes in sterol- and lipid biosynthetic pathways that may reflect altered intestinal lipid delivery, direct measurements of hepatic VLDL secretion rate, *de novo* lipogenesis, sterol synthesis flux, or related protein-level changes were not performed. Therefore, the functional significance of these transcriptional alterations in relation to the systemic lipid phenotype remains unresolved.

Fourth, intestinal Faf2 deficiency was accompanied by a substantial reduction in BW under HFD conditions, making BW and metabolic outcomes inherently linked in this model. Accordingly, the relative contributions of weight-dependent and weight-independent effects on glucose tolerance and HS could not be fully distinguished. Future studies using alternative experimental paradigms may help further clarify these relationships 48,49.

Finally, this study focused on defining the short- to mid-term metabolic consequences of intestinal Faf2 deletion. Further long-term studies may provide additional insights into intestinal safety over extended time scales.

Taken together, these findings suggest that intestinal Faf2 deficiency alters enterocyte lipid handling, ER-associated organelle homeostasis, and systemic metabolic phenotypes in mice. Intestinal Faf2 loss was associated with increased intracellular ApoB48 protein accumulation in enterocytes, enhanced LD retention, reduced CM abundance in intestinal lacteals, and dilation of the rough ER. These observations are consistent with altered intracellular lipid and lipoprotein handling in Faf2-deficient enterocytes, but do not define the precise step affected, such as ApoB48 lipidation, ER-to-Golgi trafficking, or post-ER processing. In parallel, intestinal Faf2 deficiency was accompanied by hepatic transcriptional changes involving sterol- and lipid-associated pathways, while skeletal muscle transcriptomic changes were not enriched predominantly for canonical lipid-handling pathways. Although the physiological significance and mechanistic contribution of these extra-intestinal transcriptional changes remain to be determined, the overall phenotype supports a role for intestinal Faf2 in enterocyte lipid handling and systemic metabolic homeostasis.

## Supplementary Material

Supplementary figures and tables.

## Data Availability

The RNA-seq datasets generated in this study are deposited in the Gene Expression Omnibus database.

## Abbreviations

ApoB: apolipoprotein B
BW: body weight
CM: chylomicrons
EE: energy expenditure
eGFP: enhanced green fluorescent protein
ER: endoplasmic reticulum
eWAT: epididymal white adipose tissue
Faf2: Fas-associated factor family member 2
Faf2-IKO: intestine-specific Faf2 knockout
FFA: free fatty acids
H&E: Hematoxylin and eosin
HFD: high-fat diet
HPLC: high-performance liquid chromatography
HS: hepatic steatosis
iWAT: inguinal white adipose tissue
LD: lipid droplet
LDL: low-density lipoprotein
LTT: lipid tolerance test
MTTP: microsomal triglyceride transfer protein
ND: normal diet
pWAT: periovarian white adipose tissue
RER: respiratory exchange ratio
RNA-seq: RNA-sequencing
SM: skeletal muscle
TG: triglycerides
VCO_2_: carbon dioxide production
VLDL: very low-density lipoproteins
VO_2_: oxygen consumption
WT: wild-type

## References

[1] Lee JN, Kim H, Yao H, Chen Y, Weng K, Ye J (2010). Identification of Ubxd8 protein as a sensor for unsaturated fatty acids and regulator of triglyceride synthesis. Proc Natl Acad Sci U S A.

[2] Imai N, Suzuki M, Hayashi K, Ishigami M, Hirooka Y, Abe T, Shioi G, Goto H, Fujimoto T (2015). Hepatocyte-Specific Depletion of UBXD8 Induces Periportal Steatosis in Mice Fed a High-Fat Diet. PLoS One.

[3] Suzuki M, Otsuka T, Ohsaki Y, Cheng J, Taniguchi T, Hashimoto H (2012). Derlin-1 and UBXD8 are engaged in dislocation and degradation of lipidated ApoB-100 at lipid droplets. Mol Biol Cell.

[4] Zheng J, Cao Y, Yang J, Jiang H (2022). UBXD8 mediates mitochondria-associated degradation to restrain apoptosis and mitophagy. EMBO Rep.

[5] Koyano F, Yamano K, Hoshina T (2024). AAA+ ATPase chaperone p97/VCPFAF2 governs basal pexophagy. Nature Communications.

[6] Kim C, Gabriel KR, Boone D (2025). FAF2 is a bifunctional regulator of peroxisomal homeostasis and saturated lipid responses. Science Advances.

[7] Brodsky JL, Fisher EA (2008). The many intersecting pathways underlying apolipoprotein B secretion and degradation. Trends Endocrinol Metab.

[8] Fisher EA, Ginsberg HN (2002). Complexity in the secretory pathway: The assembly and secretion of apolipoprotein B-containing lipoproteins. J Biol Chem.

[9] Ye J, Li JZ, Liu Y (2009). Cideb, an ER- and Lipid Droplet-Associated Protein, Mediates VLDL Lipidation and Maturation by Interacting with Apolipoprotein B. Cell Metabolism.

[10] Visser A, Hussain MM, Kuivenhoven JA (2025). The intracellular chylomicron highway: novel insights into chylomicron biosynthesis, trafficking, and secretion. Current Opinion in Lipidology.

[11] Siddiqi SA, Gorelick FS, Mahan JT, Mansbach CM (2003). COPII proteins are required for Golgi fusion but not for endoplasmic reticulum budding of the pre-chylomicron transport vesicle. Journal of Cell Science.

[12] Dopeso H, Rodrigues P, Cartón-García F (2024). RhoA downregulation in the murine intestinal epithelium results in chronic Wnt activation and increased tumorigenesis. iScience.

[13] Madison BB, Dunbar L, Qiao XT (2002). cis Elements of the Villin Gene Control Expression in Restricted Domains of the Vertical (Crypt) and Horizontal (Duodenum, Cecum) Axes of the Intestine. Journal of Biological Chemistry.

[14] El Marjou F, Janssen KP, Hung-Junn Chang B (2004). Tissue-specific and inducible Cre-mediated recombination in the gut epithelium. genesis.

[15] Mina AI, LeClair RA, LeClair KB (2018). CalR: A Web-Based Analysis Tool for Indirect Calorimetry Experiments. Cell Metabolism.

[16] Yao L, Seaton SC, Ndousse-Fetter S (2018). A selective gut bacterial bile salt hydrolase alters host metabolism. eLife.

[17] Andrikopoulos S, Blair AR, Deluca N, Fam BC, Proietto J (2008). Evaluating the glucose tolerance test in mice. American Journal of Physiology-Endocrinology and Metabolism.

[18] Carper D, Coué M, Laurens C, Langin D, Moro C (2020). Reappraisal of the optimal fasting time for insulin tolerance tests in mice. Molecular Metabolism.

[19] Ziouzenkova O, Ochiai M (2020). Evaluating the appropriate oral lipid tolerance test model for investigating plasma triglyceride elevation in mice. Plos One.

[20] Kim J, Kim H, Noh SH (2020). *Grasp55^-/-^* mice display impaired fat absorption and resistance to high-fat diet-induced obesity. Nature Communications.

[21] Bocian J, Jabłoński B, Nadolska-Orczyk A (2025). Validated reference genes for normalization of RT-qPCR in developing organs of wheat to study developmentally/spatio-temporally expressed family genes. Scientific Reports.

[22] Toshima G, Iwama Y, Kimura F, Matsumoto Y, Miura M, Takahashi J (2013). LipoSEARCH®; Analytical GP-HPLC method for lipoprotein profiling and its applications. J Biol Macromol.

[23] Usui S, Hara Y, Hosaki S, Okazaki M (2002). A new on-line dual enzymatic method for simultaneous quantification of cholesterol and triglycerides in lipoproteins by HPLC. Journal of Lipid Research.

[24] Ganji R, Paulo JA, Xi Y, Kline I, Zhu J, Clemen CS (2023). The p97-UBXD8 complex regulates ER-mitochondria contact sites by altering membrane lipid saturation and composition. Nat Commun.

[25] Kwon O, Han T-S, Son M-Y (2020). Intestinal Morphogenesis in Development, Regeneration, and Disease: The Potential Utility of Intestinal Organoids for Studying Compartmentalization of the Crypt-Villus Structure. Frontiers in Cell and Developmental Biology.

[26] Barker N (2013). Adult intestinal stem cells: critical drivers of epithelial homeostasis and regeneration. Nature Reviews Molecular Cell Biology.

[27] Schwantes-An TH, Darlay R, Mathurin P, Masson S, Liangpunsakul S, Mueller S (2021). Genome-wide association study and meta-analysis on alcohol-associated liver cirrhosis identifies genetic risk factors. Hepatology.

[28] Huda N, Kusumanchi P, Jiang Y, Gao H, Thoudam T, Zeng G (2025). Silencing Faf2 mitigates alcohol-induced hepatic steatosis by modulating lipolysis and PCSK9 pathway. Hepatol Commun.

[29] Bassaganya-Riera J, Xie Y, Matsumoto H (2013). Intestine-Specific Mttp Deletion Increases the Severity of Experimental Colitis and Leads to Greater Tumor Burden in a Model of Colitis Associated Cancer. PLoS ONE.

[30] Cuchel M, Meagher EA, du Toit Theron H, Blom DJ, Marais AD, Hegele RA (2013). Efficacy and safety of a microsomal triglyceride transfer protein inhibitor in patients with homozygous familial hypercholesterolaemia: A single-arm, open-label, phase 3 study. Lancet.

[31] Hussain MM, Shi J, Dreizen P (2003). Microsomal triglyceride transfer protein and its role in apoB-lipoprotein assembly. J Lipid Res.

[32] Imai N, Nicholls HT, Alves-Bezerra M, Li Y, Ivanova AA, Ortlund EA (2022). Up-regulation of thioesterase superfamily member 2 in skeletal muscle promotes hepatic steatosis and insulin resistance in mice. Hepatology.

[33] Huang X-T, Li T, Li T (2022). Embryogenic stem cell-derived intestinal crypt fission directs *de novo* crypt genesis. Cell Reports.

[34] Della Torre S, Mitro N, Fontana R (2016). An Essential Role for Liver ERα in Coupling Hepatic Metabolism to the Reproductive Cycle. Cell Reports.

[35] Yang M, Liu Q, Huang T, Tan W, Qu L, Chen T (2020). Dysfunction of estrogen-related receptor alpha-dependent hepatic VLDL secretion contributes to sex disparity in NAFLD/NASH development. Theranostics.

[36] Ganz M, Csak T, Szabo G (2014). High fat diet feeding results in gender specific steatohepatitis and inflammasome activation. World J Gastroenterol.

[37] Lonardo A, Nascimbeni F, Ballestri S (2019). Sex Differences in Nonalcoholic Fatty Liver Disease: State of the Art and Identification of Research Gaps. Hepatology.

[38] Smiriglia A, Lorito N, Serra M, Perra A, Morandi A, Kowalik MA (2023). Sex difference in liver diseases: How preclinical models help to dissect the sex-related mechanisms sustaining NAFLD and hepatocellular carcinoma. iScience.

[39] Weiss EJ, Febbraio M, Vera NB (2016). Hepatocyte-Specific Disruption of CD36 Attenuates Fatty Liver and Improves Insulin Sensitivity in HFD-Fed Mice. Endocrinology.

[40] Zhao L, Zhang C, Luo X, Wang P, Zhou W, Zhong S (2018). CD36 palmitoylation disrupts free fatty acid metabolism and promotes tissue inflammation in non-alcoholic steatohepatitis. J Hepatol.

[41] Tillander V, Alexson SEH, Cohen DE (2017). Deactivating fatty acids: Acyl-CoA thioesterase-mediated control of lipid metabolism. Trends Endocrinol Metab.

[42] Borén J, Taskinen M-R, Packard CJ (2024). Biosynthesis and Metabolism of ApoB-Containing Lipoproteins. Annual Review of Nutrition.

[43] Tong J, Tschöp MH, Aulinger BA (2010). The intestinal lymph fistula model-a novel approach to study ghrelin secretion. American Journal of Physiology-Gastrointestinal and Liver Physiology.

[44] Ko CW, Qu J, Liu M, Black DD, Tso P (2019). Use of Isotope Tracers to Assess Lipid Absorption in Conscious Lymph Fistula Mice. Current Protocols in Mouse Biology.

[45] Drucker DJ (2018). Mechanisms of Action and Therapeutic Application of Glucagon-like Peptide-1. Cell Metabolism.

[46] Miyawaki K, Yamada Y, Ban N (2017). Inhibition of gastric inhibitory polypeptide signaling prevents obesity. Nature Medicine. 2002; 8:738-742. Metab.

[47] Magkos F, Mittendorfer B (2009). Stable isotope-labeled tracers for the investigation of fatty acid and triglyceride metabolism in humans *in vivo*. Clinical Lipidology.

[48] Ravussin Y, Leibel Rudolph L, Ferrante Anthony W (2014). A Missing Link in Body Weight Homeostasis: The Catabolic Signal of the Overfed State. Cell Metabolism.

[49] Samuel VT, Shulman GI (2016). The pathogenesis of insulin resistance: integrating signaling pathways and substrate flux. Journal of Clinical Investigation.

