# Supplementary material for: Intestine-specific Faf2 depletion ameliorates obesity and metabolic dysfunction-associated steatotic liver disease by impairing lipid absorption
Source: Int J Biol Sci. 2026 Jun 10;22(12):6261–80. doi: 10.7150/ijbs.134379 (PMC13411729; doi:10.7150/ijbs.134379)
Supplement: Supplementary file 1 — Supplementary figures and tables. [file ijbsv22p6261s1.pdf]

Figure S1. Intestine-specific Faf2 depletion mice display reduced body weight and resistance to HFD-induced obesity. 4-week-old female Faf2-IKO and Control mice were fed a ND or HFD for 12 weeks. (A-C) Body weight at 4 weeks of age (A), body weight curves (B), and adult body weight (C) of female mice fed a ND or HFD (n = 6,10 for WT ND, 9,10 for Faf2-IKO ND,6 for WT HFD,6 for Faf2-IKO HFD). (D) SM-to-body weight ratio and WAT-to-body weight ratio of male mice fed a HFD (n = 6 per group). (E, F) pWAT (E) and iWAT (F) weights in female mice fed a ND or HFD (n = 10 for WT ND, 10 for Faf2-IKO ND,6 for WT HFD,6 for Faf2-IKO HFD). (G, H) H&E staining (G) and adipocyte size (H) in pWAT of female mice fed a HFD (n = 6 per group). Scale bars: 100 μm. (I) Quantitative PCR analysis of mRNA expression of adipose lipolysis–related genes in iWAT from ND-fed female Control and Faf2-IKO female mice (n = 3 per group). β-Actin was used as the internal reference gene. Data are shown as mean ± SEM. ns: not significant, *$p<0.05$, **$p<0.01$, ***$p<0.001$, ****$p<0.0001$. Statistical analyses were performed using one-way ANOVA followed by Bonferroni post-hoc testing for panels A, C, E, and F; two-way ANOVA followed by Bonferroni post-hoc testing for panel B; and multiple unpaired two-tailed Student's t-tests without correction for multiple comparisons for panels D and I. Abbreviations: Faf2, fas-associated factor family member 2; Faf2-IKO, intestine-specific Faf2 knockout; H&E, hematoxylin and eosin; HFD, high-fat diet; iWAT, inguinal white adipose tissue; LD, lipid droplet; ND, normal diet; pWAT, periovarian white adipose tissue; SM, skeletal muscle; TG, triglyceride; WT, wild type.

Figure S2. Intestine-specific Faf2 deficiency alters liver weight and glucose metabolism. 4-week-old female Faf2-IKO mice and their littermates were fed a HFD for 12 weeks. (A) Liver weight of ND-fed and HFD-fed female mice (n = 10 for WT ND, 10 for Faf2-IKO ND, 6 for WT HFD, 6 for Faf2-IKO HFD). (B) Images of H&E staining in the livers of female Faf2-IKO mice and Control mice fed ND and HFD. Scale bar: 200 μm. (C, D) Serum ALT (C) and AST (D) levels in female Control and Faf2-IKO mice fed a ND or HFD (n = 10 for WT ND, 10 for Faf2-IKO ND, 6 for WT HFD, 6 for Faf2-IKO HFD). (E, F) Glucose tolerance tests were performed on fasted (6 hours) female Faf2-IKO mice and littermates after 12 weeks of HFD. Blood glucose concentrations were monitored over a 2 h period following glucose administration (E). Bar graphs represent the AUC for all time points (F) (n = 6 for WT HFD,7 for Faf2-IKO HFD). (G, H) Insulin tolerance tests were performed on fasted (4 hours) female Faf2-IKO mice and littermates after 13 weeks of HFD. Blood glucose concentrations were measured 2 hours after the insulin injection (G). Bar graphs represent the AUC for all time points (H) (n = 6 for WT HFD,8 for Faf2-IKO HFD). Data are shown as mean ± SEM. ns: not significant, *$p<0.05$, **$p<0.01$, ***$p<0.001$. Statistical analyses were performed using one-way ANOVA followed by Bonferroni post-hoc testing for panels A, C, and D; two-way ANOVA followed by Bonferroni post-hoc testing for panels E and G; and unpaired two-tailed Student's t-tests for panels F and H. Abbreviations: ALT, alanine aminotransferase; AST, aspartate aminotransferase; AUC, area under the curve; Faf2, fas-associated factor family member 2; Faf2-IKO, intestine-specific Faf2 knockout; H&E, hematoxylin and eosin; HFD, high-fat diet; ND, normal diet; WT, wild type.

Figure S3. Metabolic profiles of Faf2-IKO female mice. (A) Analyses of oxygen consumption, (B) carbon dioxide production, (C) respiratory exchange ratio, (D) energy expenditure, (E) locomotor activity, and (F) food intake in 12-week-old female Faf2-IKO mice and littermates fed a ND (n = 6 per group). (G) Summary of ANCOVA and ANOVA statistical analyses for each metabolic parameter. (H) Fecal caloric content was measured using bomb calorimetry in female Faf2-IKO and Control mice (n = 7 for WT ND,6 for Faf2-IKO ND). Data are shown as mean ± SEM. ns: not significant, *p<0.05, **p<0.01. Statistical analyses were performed using two-way ANOVA for C, E, ANCOVA for A, B, D, F, unpaired two-tailed Student's t test for H. Abbreviations: Faf2, fas-associated factor family member 2; Faf2-IKO, intestine-specific Faf2 knockout; ND, normal diet.

Figure S4. Faf2 is essential for efficient intestinal lipid metabolism. (A-D) Plasma levels of TG (A), FFA (B), phospholipids (C), and total cholesterol (D) were measured in female mice fed ND or HFD (n = 10 for WT ND, 10 for Faf2-IKO ND, 6 for WT HFD, 6 for Faf2-IKO HFD). Data are shown as mean ± SEM. ns: not significant, *$p<0.05$, **$p<0.01$, ***$p<0.001$. Statistical analyses were performed using one-way ANOVA followed by Bonferroni post-hoc testing for panels A–D. Abbreviations: Faf2, fas-associated factor family member 2; Faf2-IKO, intestine-specific Faf2 knockout; FFA, free fatty acids; HFD, high-fat diet; ND, normal diet; TG, triglycerides.

Figure S5. Intestinal morphology and epithelial proliferation are unaltered in Faf2-IKO male mice. (A) H&E-stained slides of the jejunum from Control and Faf2-IKO male mice at 4-week-old, crypt-villus junctions were established (dotted line) and villus height and crypt depth (brackets). Scale bars: 100 μm (B) Villus length and crypt length were measured using Image J software, while villus and crypt cell numbers were determined by counting the visible nuclei in the epithelial layer in mice fed a ND or HFD (crypts > 20 or villi > 20 from 4 mice per group). (C, D) Representative immunohistochemical staining for Ki67 in the jejunum of ND-fed Control and Faf2-IKO male mice (C). Arrowheads indicate Ki67-positive cells in the crypt region. Insets show higher-magnification views (n= 4 per group). Quantification of Ki67-positive cells in the jejunum, expressed as the percentage of Ki67-positive epithelial cells (D). Data are shown as mean ± SEM. ns: not significant. Statistical analyses were performed using unpaired two-tailed Student's t-tests for panels B and D. Abbreviations: Faf2, fas-associated factor family member 2; Faf2-IKO, intestine-specific Faf2 knockout; H&E, hematoxylin and eosin; ND, normal diet.

Figure S6. Faf2 depletion affects lipid absorption in the small intestine. (A) The lengths of the colon and small intestine were measured in female mice fed an ND or HFD. Small intestine length was defined as the distance from the ligament of Treitz to the cecum (n = 9 for WT ND, 6,8 for Faf2-IKO ND, 6 for WT HFD, 6 for Faf2-IKO HFD). (B) H&E-stained slides of the jejunum from Control and Faf2-IKO female mice, crypt-villus junctions (dotted line) and villus height and crypt depth (brackets). Scale bars: 100 μm. (C) Villus length and crypt length were measured using Image J software, while villus and crypt cell numbers were determined by counting the visible nuclei in the epithelial layer in mice fed a ND or HFD (n > 18 crypts or n > 18 villi from 3-4 mice per group). (D, E) Representative immunohistochemical staining for Ki67 in the jejunum of ND-fed Control and Faf2-IKO female mice (D). Arrowheads indicate Ki67-positive cells in the crypt region. Insets show higher-magnification views (n= 4 per group). Quantification of Ki67-positive cells in the jejunum, expressed as the percentage of Ki67-positive epithelial cells (E). Data are shown as mean ± SEM. ns: not significant, ***p<0.001, ****p<0.0001. Statistical analyses were performed using one-way ANOVA followed by Bonferroni post-hoc testing for panels A and C, and an unpaired two-tailed Student's t-test for panel E. Abbreviations: Faf2, fas-associated factor family member 2; Faf2-IKO, intestine-specific Faf2 knockout; H&E, hematoxylin and eosin; HFD, high-fat diet; ND, normal diet; WT, wild type.

Figure S7. Intestinal Faf2 deficiency is associated with Rough ER dilation and altered expression of genes related to organelle homeostasis in jejunal enterocytes. (A) Representative transmission electron micrographs of jejunal enterocytes from ND-fed male Control and Faf2-IKO mice. Mice were fasted for fasted for 16 h and analyzed 2 h after oral gavage of olive oil (10 μL/g body weight). Orange arrowheads indicate Rough ER. Images were acquired at ×30,000 magnification. Scale bars: 1 μm. (B) Quantification of Rough ER width in jejunal enterocytes from ND-fed male Control and Faf2-IKO mice. For each mouse, three independent transmission electron micrographs images were analyzed, and 20 Rough ER width measurements were obtained per image (n = 3 per group). (C) Quantitative PCR analysis of genes related to organelle homeostasis, ER stress, and intracellular lipid handling in the jejunum of ND-fed male Control and Faf2-IKO mice (n = 4 per group). β-Actin was used as the internal control. Data are shown as mean ± SEM. ns: not significant, *p<0.05, **p<0.01， ****p<0.0001. Statistical analyses were performed using an unpaired two-tailed Student's t-test for panel B and multiple unpaired two-tailed Student's t-tests without correction for multiple comparisons for panel C. Abbreviations: ER, endoplasmic reticulum; Faf2, fas-associated factor family member 2; Faf2-IKO, intestine-specific Faf2 knockout; ND, normal diet.

Figure S8. Intestinal Faf2 deficiency alters jejunal expression of genes related to lipid and glucose handling before and after the LTT. (A) Quantitative PCR analysis of genes related to fatty acid transport, glucose transport, and nuclear receptor signaling in the jejunum of ND-fed male Control and Faf2-IKO mice (n = 3 or 4 per group). Cyclophilin A was used as the internal control. (B) Quantitative PCR analysis of genes related to fatty acid transport, glucose transport, and nuclear receptor signaling in the jejunum of ND-fed male Control and Faf2-IKO mice following LTT (n = 4 per group). Cyclophilin A was used as the internal control. Data are shown as mean ± SEM. ns: not significant, *$p<0.05$, **$p<0.01$, ***$p<0.001$. Statistical analyses were performed using multiple unpaired two-tailed Student's t-tests without correction for multiple comparisons for panels A and B. Abbreviations: Faf2, fas-associated factor family member 2; Faf2-IKO, intestine-specific Faf2 knockout; LTT, lipid tolerance test; mRNA, messenger RNA; ND, normal diet.

Figure S9. Intestinal Faf2 deficiency alters plasma FFA and TG levels during LTT. (A, B) Plasma FFA levels (A) and AUC (B) in Faf2-IKO female ND-fed mice and Controls following a LTT with olive oil gavage (10 μL/g body weight) after 16 h fasting (n = 7 per group). (C, D) Plasma TG levels (C) and AUC (D) in the same experimental setting as in Faf2-IKO mice (n = 6 per group). Data are shown as mean ± SEM. ns: not significant, *p<0.05, **p<0.01, ****p<0.0001. Statistical analyses were performed using two-way ANOVA followed by Bonferroni post-hoc testing for panels A and C, and unpaired two-tailed Student's t-tests for panels B and D. Abbreviations: AUC, area under the curve; Faf2, fas-associated factor family member 2; Faf2-IKO, intestine-specific Faf2 knockout; FFA, free fatty acids; LTT, lipid tolerance test; ND, normal diet; TG, triglycerides.

Figure S10. Transcriptomic profiles of skeletal muscles in Faf2-IKO male mice. (A) Volcano plot showing differential gene expression in the skeletal muscles of Faf2-IKO versus control mice. Significantly upregulated (red) and downregulated (blue) genes are indicated (cutoff: $|\log_2 FC| > 2$, FDR < 0.05). (B) Heatmap of the top differentially expressed genes across groups clustered by expression patterns. (C) GO enrichment analysis of the differentially expressed genes. The top pathways enriched in BP (green), MF (red), and CC (blue) are shown. Although significant GO terms were identified, no major metabolic pathways were found to be significantly altered (n = 4 per group). Abbreviations: BP, biological processes; CC, cellular components; Faf2, fas-associated factor family member 2; Faf2-IKO, intestine-specific Faf2 knockout; GO, Gene Ontology; MF, molecular functions; MHC, major histocompatibility complex.

Figure S1

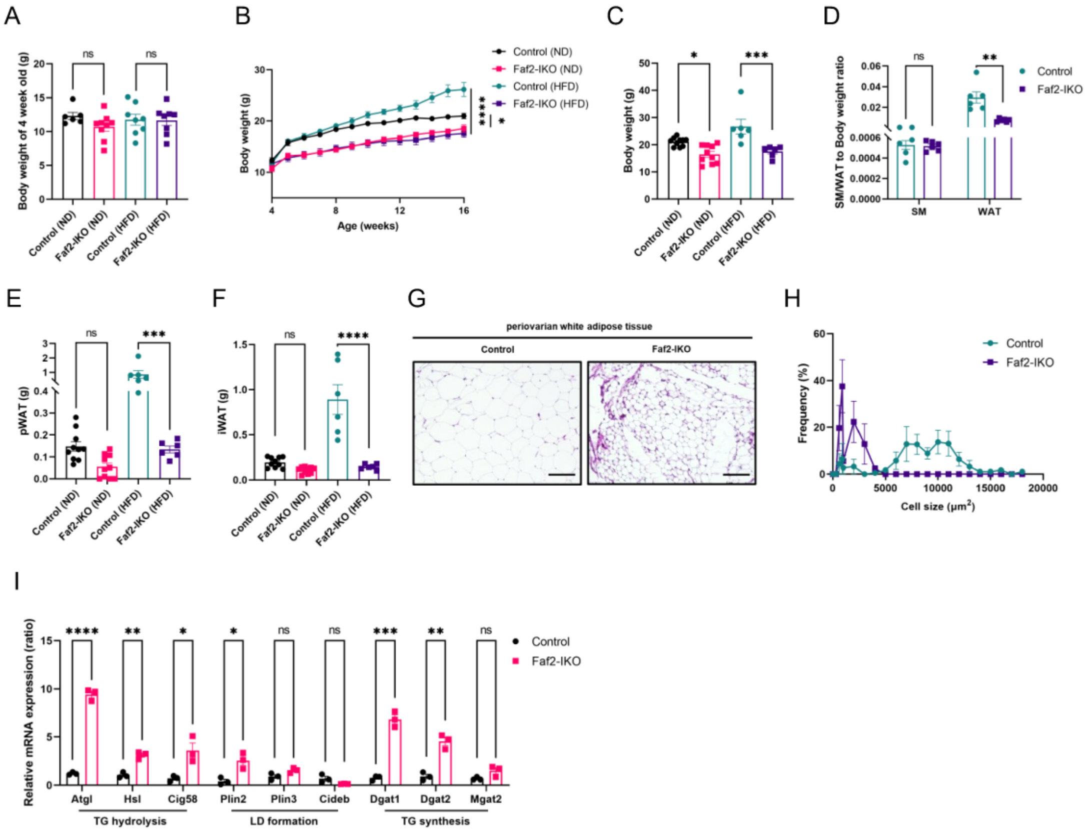

A
B
C
D
E
F
G
H
I
Body weight of 4 week old (g)
Control (ND)
Faf2-IKO (ND)
Control (HFD)
Faf2-IKO (HFD)
Body weight (g)
Age (weeks)
Control
Faf2-IKO
SM/WAT to Body weight ratio
SM
WAT
pWAT (g)
iWAT (g)
periovarian white adipose tissue
Frequency (%)
Cell size (μm²)
Relative mRNA expression (ratio)
Atgl
Hsl
Cig58
Plin2
Plin3
Cideb
Dgat1
Dgat2
Mgat2
TG hydrolysis
LD formation
TG synthesis

Figure S2

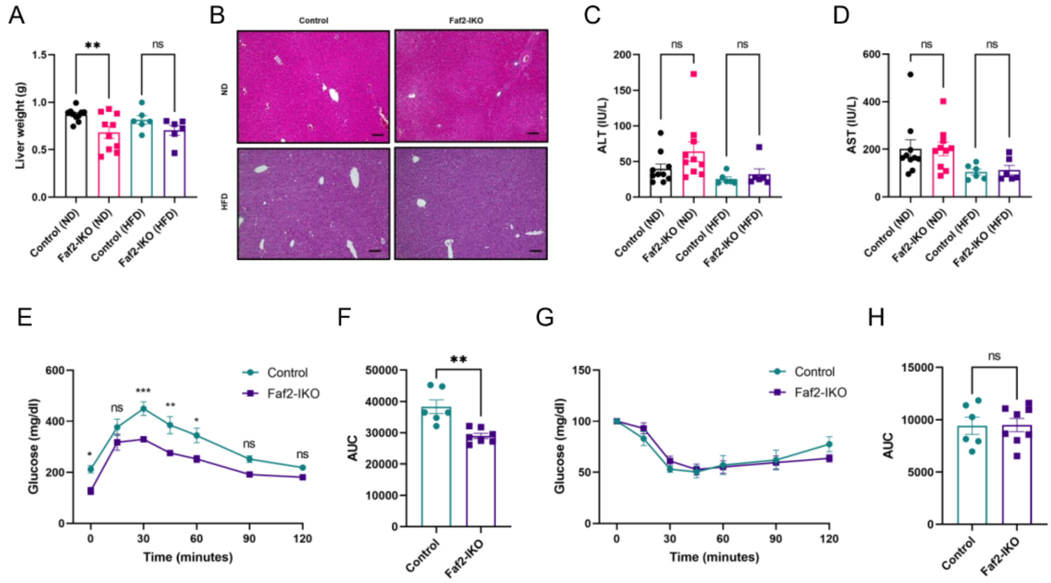

A
B
C
D
E
F
G
H
Liver weight (g)
Control (ND)
Faf2-IKO (ND)
Control (HFD)
Faf2-IKO (HFD)
Control
Faf2-IKO
ND
HFD
ALT (IU/L)
AST (IU/L)
Glucose (mg/dl)
Time (minutes)
AUC
ns

Figure S3

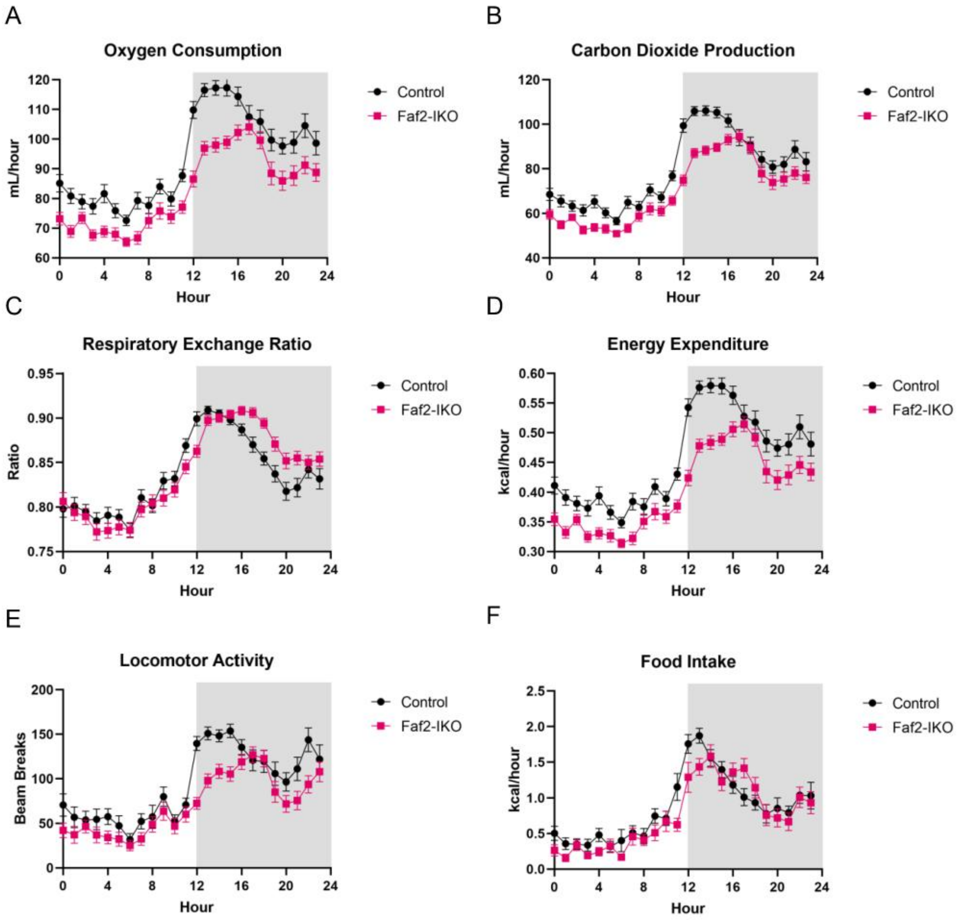

G

Mass-dependent

	Full Day	Light	Dark
Food Consumed (kcal/period)	0.9318	0.1790	0.6931
Water Consumed (ml/period)	0.0378 *	0.0027 **	0.0991
Energy Expenditure (kcal/period)	0.0346 *	0.0161 *	0.0742
Oxygen Consumption (ml/hr)	0.0332 *	0.0153 *	0.0688
Carbon Dioxide Production (ml/hr)	0.0436 *	0.0244	0.1024

Mass-independent

	Full Day	Light	Dark
Pedestrian Locomotion (m)	0.1537	0.1598	0.1483
Total Distance in Cage (m)	0.1345	0.1414	0.1301
Respiratory Exchange Ratio	0.8318	0.5614	0.0773
Locomotor Activity (beam breaks)	0.0499 *	0.2015	0.0380 *
Energy Balance (kcal/period)	0.7475	0.2661	0.9570

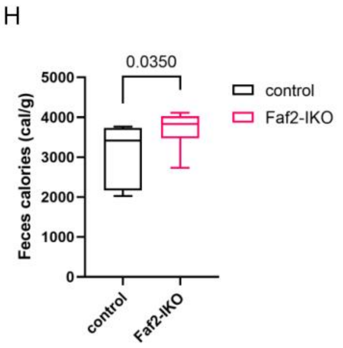

Figure S4

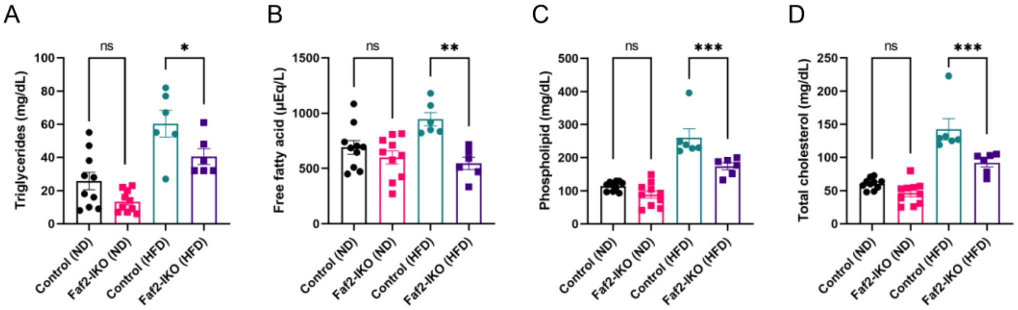
A
B
C
D
Triglycerides (mg/dL)
Free fatty acid (μEq/L)
Phospholipid (mg/dL)
Total cholesterol (mg/dL)
ns
*
**
***
Control (ND)
Faf2-IKO (ND)
Control (HFD)
Faf2-IKO (HFD)

Figure S5

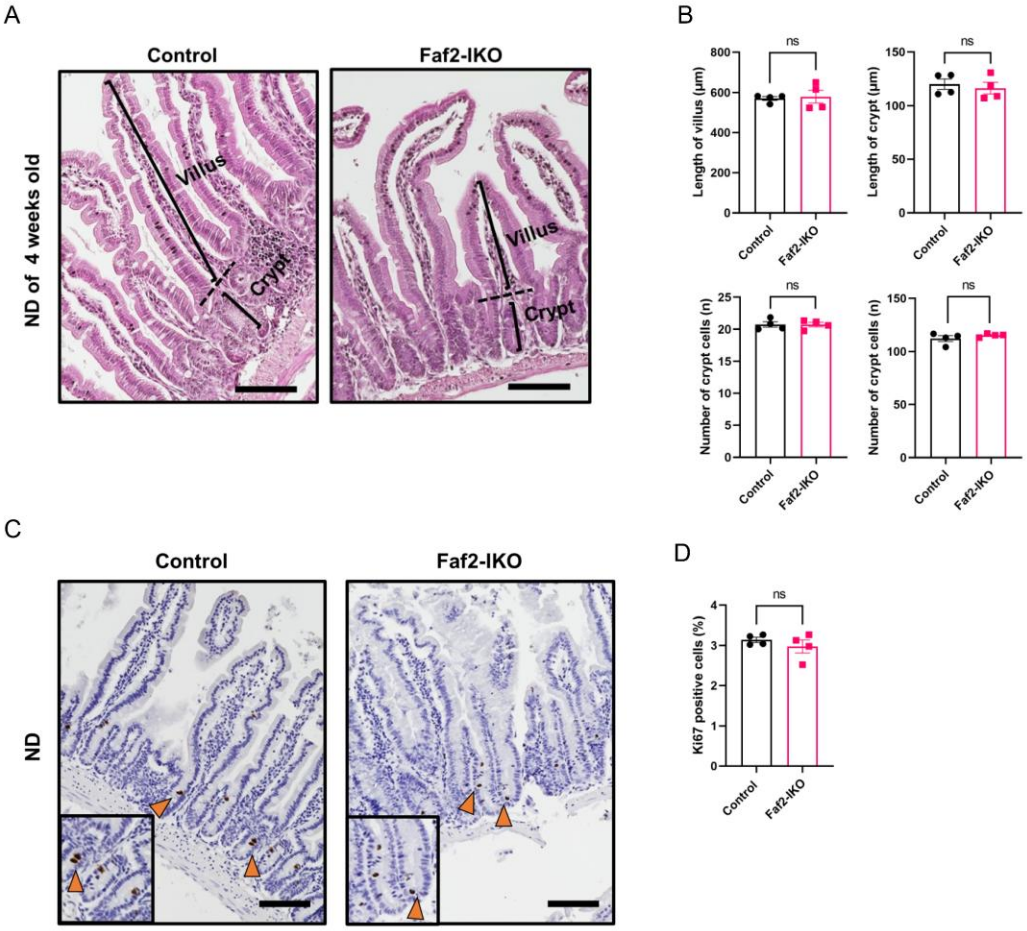
A
Control
Faf2-IKO
ND of 4 weeks old
Villus
Crypt
Villus
Crypt
B
ns
Length of villus (µm)
Length of crypt (µm)
Number of crypt cells (n)
Number of crypt cells (n)
Control
Faf2-IKO
C
Control
Faf2-IKO
ND
D
ns
Ki67 positive cells (%)
Control
Faf2-IKO

Figure S6

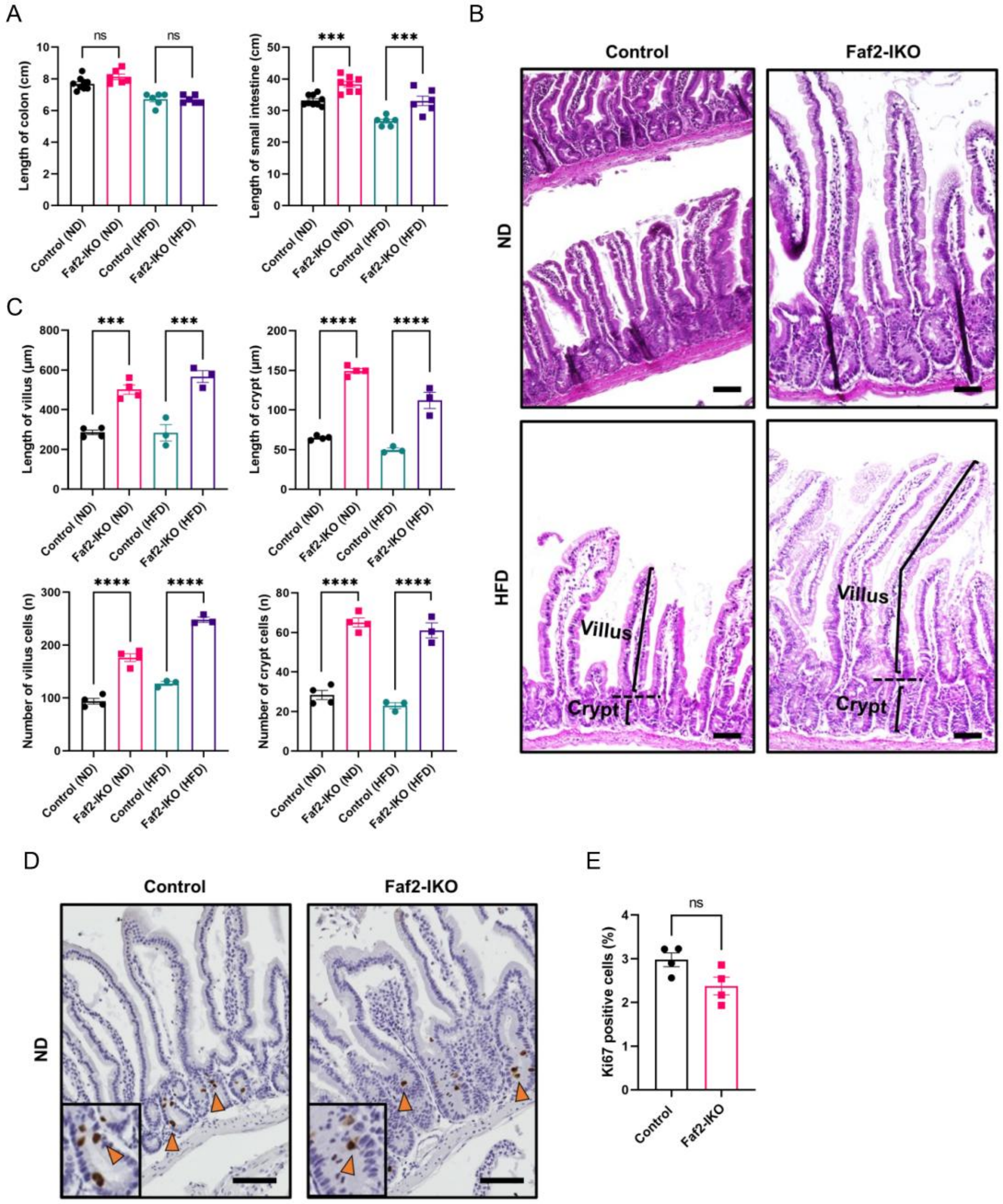

A
ns
ns
***
***
Length of colon (cm)
Length of small intestine (cm)
Control (ND)
Faf2-IKO (ND)
Control (HFD)
Faf2-IKO (HFD)
B
Control
Faf2-IKO
ND
HFD
Villus
Crypt
C
****
Length of villus (μm)
Length of crypt (μm)
Number of villus cells (n)
Number of crypt cells (n)
D
Control
Faf2-IKO
ND
E
Ki67 positive cells (%)
Control
Faf2-IKO

Figure S7

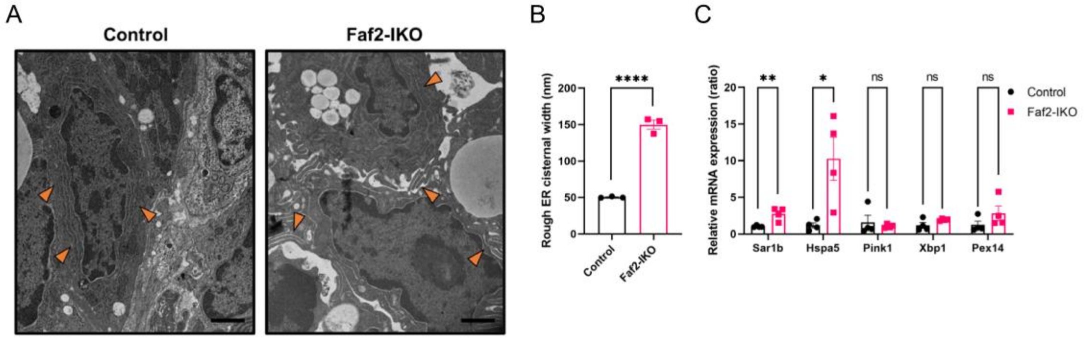
A
Control
Faf2-IKO
B
Rough ER cisternal width (nm)
****
Control
Faf2-IKO
C
Relative mRNA expression (ratio)
**
*
ns
ns
ns
Sar1b
Hspa5
Pink1
Xbp1
Pex14
Control
Faf2-IKO

Figure S8

A

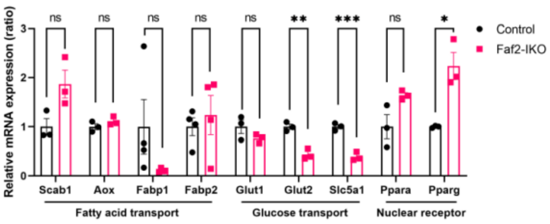

Relative mRNA expression (ratio)
Control
Faf2-IKO
ns ns ns ns ns ** *** ns *
Scab1 Aox Fabp1 Fabp2 Glut1 Glut2 Slc5a1 Ppara Pparg
Fatty acid transport
Glucose transport
Nuclear receptor

B

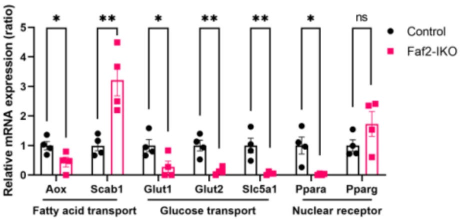

Relative mRNA expression (ratio)
Control
Faf2-IKO
* ** * ** ** * ns
Aox Scab1 Glut1 Glut2 Slc5a1 Ppara Pparg
Fatty acid transport
Glucose transport
Nuclear receptor

Figure S9

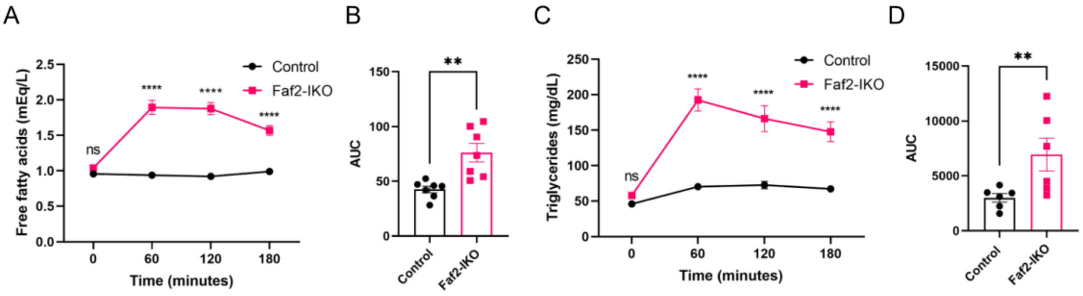
A
Free fatty acids (mEq/L)
Time (minutes)
Control
Faf2-IKO
ns
****
B
AUC
**
Control
Faf2-IKO
C
Triglycerides (mg/dL)
Time (minutes)
Control
Faf2-IKO
ns
****
D
AUC
**
Control
Faf2-IKO

Figure S10

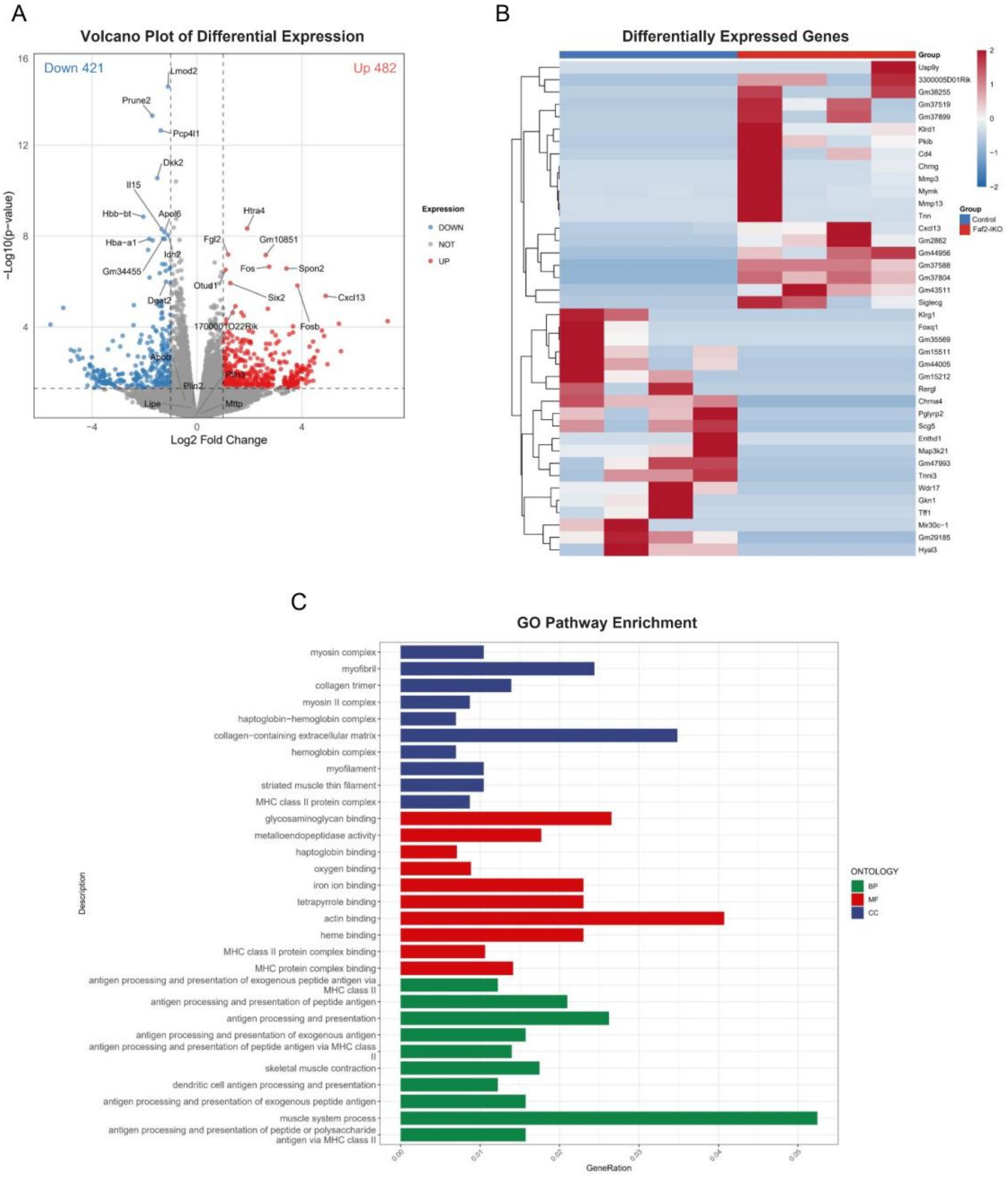
A
Volcano Plot of Differential Expression
Down 421
Up 482
-Log10(p-value)
Log2 Fold Change
Expression
DOWN
NOT
UP
Lmod2
Prune2
Pcp4l1
Dkk2
Il15
Hbb-bt
Apol6
Hba-a1
Idh2
Gm34455
Dgat2
Apob
Plin2
Lipe
Htra4
Gm10851
Fgl2
Fos
Spon2
Otud1
Six2
Cxcl13
1700001O22Rik
Fosb
Mttp
B
Differentially Expressed Genes
Group
Control
Faf2-IKO
C
GO Pathway Enrichment
myosin complex
myofibril
collagen trimer
myosin II complex
haptoglobin−hemoglobin complex
collagen−containing extracellular matrix
hemoglobin complex
myofilament
striated muscle thin filament
MHC class II protein complex
glycosaminoglycan binding
metalloendopeptidase activity
haptoglobin binding
oxygen binding
iron ion binding
tetrapyrrole binding
actin binding
heme binding
MHC class II protein complex binding
MHC protein complex binding
antigen processing and presentation of exogenous peptide antigen via MHC class II
antigen processing and presentation of peptide antigen
antigen processing and presentation
antigen processing and presentation of exogenous antigen
antigen processing and presentation of peptide antigen via MHC class II
skeletal muscle contraction
dendritic cell antigen processing and presentation
antigen processing and presentation of exogenous peptide antigen
muscle system process
antigen processing and presentation of peptide or polysaccharide antigen via MHC class II
Description
GeneRatio
ONTOLOGY
BP
MF
CC

Table S1: Nucleotide sequences of Faf2-IKO mice

Name	Forward Primer	Reverse Primer
Faf2	GCTGCAGTTTCAGGTAGCAGCGAGTTCT	CCAACTCTGCGATTGGGGATCTGAGTTA
Villin-Cre	CATGTCCATCAGGTTCTTGC	TTCTCCTCTAGGCTCGTCCA
Villin-Cre-PC	CTAGGCCACAGAATTGAAAGATCT	GTAGGTGGAAATTCTAGCATCATCC

Table S2: Antibodies used in this study

Antibody/host	Source/catalogue number/dilution
Faf2/Rabbit	Genetex/GTX115680/WB: 1:1000
eGFP/Rabbit	Novus Biological/NB100-1678/IF: 1:500
ApoB/Rabbit	Meridian Bioscience/WB: 1:500
HSP90/Mouse	Santa Cruz Biotechnology/ sc-13119 /WB: 1:1000
StarBright Blue 520 Goat Anti-Mouse IgG	BIORAD/12005867/ WB: 1:1000
Ki67	Abcam/ab16667/IHC: 1:100
Goat anti-Rabbit IgG	Vector Laboratories/ MP-7404-50/IHC: 1:100
Alexa Fluor 647/Goat	Invitrogen/A-21244/IF: 1:300
Anti-Actin hFAB Rhodamine	BIORAD/12004164/WB: 1:1000

Table S3: qPCR primers used in this study

Origin	Gene Name	Forward Primer	Reverse Primer
Mouse	Faf2	GAGCAGGATCTAACTCAGGAGC	CAGCAGCCTCCATGTTCCAG
Mouse	Cd36	CAACCACTGTTTCTGCACTG	CAGGCTTTCCTTCTTTGCAC
Mouse	Fatp4	TATGGCTTCCCTGGTGTACTAT	TTCTTCCGGATCACCACAGTCA
Mouse	Fabp1	GGTGACAACTTTCAAAGGCATAAA	TGTCGCCCAATGTCATGGTA
Mouse	Fabp2	CTCGGTGTAAACTTTCCCTACAGTC	TTTATTTCCCTCAATGGTCCAG
Mouse	Fabp6	CTTCCAGGAGACGTGATTGAAA	CCTCCGAAGTCTGGTGATAGTTG
Mouse	Npc1l1	CGCCCTTCTTTCTACATGGGT	GAATCTGCGCTTACGAGGGAG
Mouse	Scab1	TTTGGAGTGGTAGTAAAAAGGGC	TGACATCAGGGACTCAGAGTAG
Mouse	Mgat2	CCTTCGCGGTCCTTCAGT	GAGGCCTACGAAGATGACGAT
Mouse	Dgat1	GGCCCAAGGTAGAAGAGGAC	GATCAGCATCACCACACACC
Mouse	Dgat2	AGGATCTGCCCTGTCACG	GCCAGCCAGGTGAAGTAGAG
Mouse	Plin2	GGAGTGGAAGAGAAGCAT	CAACACAGTGGGACTCATG
Mouse	Plin3	ATGAACACTCCCTCGGCAAG	GATTCCATCAGGCCGAGCA
Mouse	Cideb	TTGGCAGCCTCAACGTGAAA	AGGAGCTCCCTGAGTACTCTTT
Mouse	Atgl	AACACCAGCATCCAGTTCAA	GGTTCAGTAGGCCATTCCTC
Mouse	Cgi58	CCTCCCAGCGACCCTTTATC	AGGTGGTGAGAACAAGCACT
Mouse	Hsl	TGTGGCTTGCGCTCTGG	CCGCGCGAACATGACC
Mouse	ApoB	TACTTCCACCCACAGTCCCCT	GGAAGCTGCCTCTTCTTCCCAA
Mouse	Mtp	GGTGAAAGGAACCCAAGCAA	CTTCCCATATACACCAGAAGGGG
Mouse	Aox	TCAACAGCCCACTGTGACTTCCATTA	TCAGGTAGCATTATCCATCTCTCA
Mouse	Glut1	CAATGAAGTTTGAGGTCCAGTTGG	TGTGGTGTCGCTGTTTGTTGTAG
Mouse	Glut2	GACATCGGTGTGATCAATGC	CAGGGAGCTGGTGTTGTGTA
Mouse	Slc5a1	ATGCGGCTGACATCTCAGTC	ACCAAGGCGTTCCATTCAAAG
Mouse	Fxra	CCGAGAGAAGAACCGAGTT	TAGATGCCAGGAGAATACCAG
Mouse	Ppara	GTGGCTGCTATAATTTGCTGTG	GAAGGTGTCATCTGGATGGTT
Mouse	Ppard	TCCATCGTCAACAAAGACGGG	ACTTGGGCTCAATGATGTCAC
Mouse	Pparg	CACAATGCCATCAGGTTTGG	GCTGGTCGATATCACTGGAGATC
Mouse	Sar1b	CATTGCCAACGTGCCTATTCT	ACCAAACATCTCTCGTAACCTCT
Mouse	Hspa5	GACTGCTGAGGCGTATTTGG	AGCATCTTTGGTTGCTTGTCG
Mouse	Xbp1	AGCTTTTACGGGAGAAAACTCAC	CCTCTGGAACCTCGTCAGGA
Mouse	Pink1	CGACAACATCCTTGTGGAGTGG	CATTGCCACCACGCTCTACACT
Mouse	Pex14	ACAGCAGTGAAGTTCCTACAGA	GCCAGGTCAATCTCTTCGTCT
Mouse	cyclophilin A	GGCCGATGACGAGCCC	TGTCTTTGGAACTTTGTCTGCAA
Mouse	β-Actin	AGAGGGAAATCGTGCGTGAC	CAATAGTGATGACCTGGCCGT
